# Associations of dietary indices with risk of all-cause and cardiovascular mortality in hypertensive adults

**DOI:** 10.1080/07853890.2025.2584427

**Published:** 2025-11-15

**Authors:** Feng Chen, Hao Lin, Yifan Shen, Longyu Fang, Xiaoli Chen, Daishan Zheng, Mengchen Sun, Yuansi Zhang, Maoping Chu, Xiufeng Huang

**Affiliations:** ^a^ Children’s Heart Center, Institute of Cardiovascular Development and Translational Medicine, The Second Affiliated Hospital and Yuying Children’s Hospital of Wenzhou Medical University, Wenzhou, Zhejiang, China; ^b^ Zhejiang Provincial Clinical Research Center for Pediatric Precision Medicine, The Second Affiliated Hospital and Yuying Children’s Hospital of Wenzhou Medical University, Wenzhou, Zhejiang, China; ^c^ Department of Child Healthcare, Wenzhou People’s Hospital, Wenzhou, Zhejiang, China; ^d^ Department of Gastroenterology, Pingyang Hospital of Wenzhou Medical University, Wenzhou, Zhejiang, China; ^e^ Department of Pediatrics, the Second School of Medicine, The Second Affiliated Hospital and Yuying Children’s Hospital of Wenzhou Medical University, Wenzhou, Zhejiang, China; ^f^ Department of Traditional Chinese Medicine, Wenzhou Yebo Proctology Hospital, Wenzhou, Zhejiang, China

**Keywords:** Dietary patterns, hypertension, inflammation, mortality, survival analysis

## Abstract

**Background:**

Dietary patterns play a pivotal role in managing hypertension and preventing mortality. However, comparative studies on the effects of different dietary patterns on mortality risk in hypertensive patients remain limited. The aim of this study is to evaluate and compare the associations between six established dietary indices and mortality risk in this population.

**Methods:**

Utilizing data from the 2005–2018 NHANES, 13,230 hypertensive adults were included in the analysis. Six standardized dietary indices were employed, and the associations between these dietary patterns and mortality were analyzed using a weighted Cox proportional hazards model. Additionally, time trend analysis was conducted to examine changes in dietary patterns, while weighted quantile regression (WQS) was used to identify key dietary components contributing to mortality risk.

**Results:**

Over a median follow-up of 8.3 years, a total of 2,420 deaths were recorded, including 637 due to cardiovascular diseases. Higher scores for AHEI, DASH, HEI-2020, MED, and MEDI were significantly associated with reduced risk of all-cause mortality, whereas a proinflammatory diet, reflected by elevated DII scores, was associated with increased risk. Only higher DASH index scores were independently associated with reduced cardiovascular mortality. Time trend analysis revealed a decline in adherence to DASH over the years, whereas MEDI scores slightly increased. WQS regression identified dairy products, whole grains, and fatty acids as key dietary components influencing mortality risk.

**Conclusions:**

Healthy dietary patterns markedly reduce all-cause mortality risk in hypertensive patients, with the DASH diet offering notable cardiovascular protection. These findings support personalized dietary interventions for hypertension management.

## 1. Introduction

Hypertension is one of the leading chronic diseases worldwide and poses a significant challenge to public health. According to a report published by the World Health Organization (WHO) in 2023, the number of individuals aged 30–79 years with hypertension has reached 1.28 billion, affecting one-third of adults worldwide [1]. This prevalent and potentially fatal condition can lead to stroke, heart attack, heart failure, kidney damage, and even death [2–4]. Consequently, understanding treatment strategies and preventive measures for hypertension has become a critical task in the global public health arena.

In addition to its role as a hemodynamic disorder, hypertension has increasingly been recognized as a condition characterized by chronic low-grade inflammation. Inflammatory processes contribute to endothelial dysfunction and vascular remodeling, both of which are central to hypertension pathogenesis [5]. Moreover, elevated levels of inflammatory markers are independently associated with increased risks of all-cause, cardiovascular, and cancer mortality [6]. These findings underscore the importance of addressing inflammation as a key mechanism underlying adverse outcomes in hypertensive individuals.

In this context, diet has emerged as a modifiable lifestyle factor that not only influences blood pressure but also affects systemic inflammation. Certain dietary components, particularly those rich in fiber, antioxidants, and healthy fats, may help suppress inflammatory responses, whereas others, such as processed sugars and saturated fats, may exacerbate them [7]. Therefore, evaluating dietary patterns from both cardiovascular and inflammatory perspectives offers a more holistic understanding of their impacts on long-term health outcomes.

Building upon this, increasing evidence suggests that specific dietary patterns can significantly influence health outcomes in hypertensive patients. Unlike traditional analyses that focus on single nutrients or foods, dietary pattern scores provide a more comprehensive reflection of an individual’s overall dietary quality. Therefore, conducting comparative studies using multiple scoring systems not only helps identify key dietary components but also clarifies the explanatory power and sensitivity differences of various scores across different health outcomes. For example, the Dietary Approaches to Stop Hypertension (DASH) diet, which is rich in fruits, vegetables, whole grains, and low-fat dairy products, has been repeatedly shown to significantly reduce blood pressure [8,9]. Moreover, the Mediterranean Diet (MED), characterized by a high intake of olive oil, nuts, fish, and vegetables, has also been demonstrated to be beneficial for cardiovascular health [10–13]. In addition to these two dietary patterns, composite dietary quality assessment tools such as the Alternative Healthy Eating Index (AHEI) and the Healthy Eating Index 2020 (HEI-2020) have been widely applied in recent years to investigate the impact of diet on cardiovascular disease risk, revealing strong associations with cardiovascular health [14,15].

Although the literature has revealed the relationships between various dietary patterns and the incidence of hypertension and its complications, research on the long-term impacts of these dietary patterns on mortality—particularly all-cause and cardiovascular mortality in hypertensive patients—remains limited. In addition, few studies have examined how adherence to these dietary patterns has changed over time in this population and how these trends may be shaped by structural factors such as food access, economic disparities, and nutrition literacy. Therefore, clarifying the long-term effects of different dietary patterns on mortality in hypertensive patients, in conjunction with their evolving adherence trajectories, is of significant clinical and public health importance. Therefore, the present study aims to fill this gap by (1) assessing temporal trends in adherence to several major dietary patterns—including the AHEI, DASH, DII, HEI-2020, MED, and MEDI—in hypertensive U.S. adults and (2) examining the associations of these patterns with all-cause and cardiovascular mortality. Through this research, we seek to provide updated, evidence-based insights to inform long-term dietary management and public health recommendations for individuals with hypertension.

## 2. Materials and methods

### 2.1. Study population

The National Health and Nutrition Examination Survey (NHANES) is a nationally representative cross-sectional program designed to assess the health and nutritional status of the noninstitutionalized U.S. population. The NHANES is conducted by the Centers for Disease Control and Prevention (CDC) and uses a complex, multistage probability sampling design to ensure robust demographic and clinical representativeness. Data collection included structured interviews, physical examinations, and laboratory tests, with protocols approved by the National Center for Health Statistics (NCHS) Institutional Review Board. All participants provided written informed consent. In this study, we utilized data from seven NHANES cycles conducted between 2005 and 2018. To align with the objectives of the study, we applied specific inclusion criteria and the following exclusion standards: 1) exclusion of participants under the age of 20 (*n* = 30,441); 2) exclusion of pregnant participants (*n* = 566); 3) exclusion of participants with missing dietary data (*n* = 8,783); 4) exclusion of participants with missing hypertension information or those for whom hypertension could not be defined (*n* = 17,155); and 5) exclusion of participants with missing mortality data (*n* = 15). Ultimately, a total of 13,230 eligible participants were included in the study (Figure S1).

### 2.2. Definition of hypertension

Hypertension was defined on the basis of the integration of multiple criteria. After resting quietly for 5 min, the participants had their blood pressure measured by trained health care professionals using standardized methods, with the final assessment based on the average of all valid readings. Hypertension was diagnosed if the participants had an average systolic blood pressure (SBP) ≥140 mmHg or an average diastolic blood pressure (DBP) ≥90 mmHg, were receiving antihypertensive treatment, or self-reported a physician diagnosis of hypertension.

### 2.3. Assessment of dietary indices

In this study, six commonly used dietary indices (scoring systems) were applied to assess the dietary habits of hypertensive individuals. These include AHEI, DASH, DII, HEI-2020, MED, and MEDI. A detailed description of each dietary index, including the components and scoring systems, is provided in the Supplementary Materials. These dietary indices serve as key exposure variables for assessing mortality risk in hypertensive populations.

### 2.4. Mortality outcomes

Mortality data were obtained from the National Death Index (NDI) through linkage with NHANES participant records (2005–2018). The NCHS determined vital status and cause of death, coded according to the International Classification of Diseases, Tenth Revision (ICD-10). The primary outcome was all-cause mortality, and the secondary outcome was cardiovascular disease (CVD) mortality (ICD-10 codes: I00–I09, I11, I13, I20–I51, I60–I69). The follow-up time was calculated from the baseline examination date to the date of death or the end of follow-up (December 31, 2018), whichever occurred first. Participants without a death record were censored at the end of the study period.

### 2.5. Assessment of covariates

In this study, we systematically evaluated potential confounders by including demographic, lifestyle, clinical, and biochemical covariates on the basis of literature and clinical relevance [16,17]. The demographic factors included age, sex, race/ethnicity, socioeconomic status (education level, family income–poverty ratio [PIR]), and marital status. Lifestyle-related variables included smoking status (never, former, or current smoker), body mass index (BMI), waist circumference, and total energy intake. Clinical conditions were strictly defined: CVD included coronary artery disease, congestive heart failure, myocardial infarction, angina, or a history of stroke; diabetes was categorized on the basis of fasting glucose ≥7.0 mmol/L, HbA1c ≥6.5%, random glucose ≥11.1 mmol/L, self-reported diagnosis, or use of antidiabetic medications; chronic kidney disease (CKD) was defined by an estimated glomerular filtration rate (eGFR) <60 mL/min/1.73 m^2^ (calculated using the CKD–EPI equation); dyslipidemia was defined as total cholesterol ≥200 mg/dL, triglycerides ≥150 mg/dL, HDL-*C* < 40 mg/dL (men) or <50 mg/dL (women), LDL-*C* ≥ 130 mg/dL, or lipid-lowering therapy; and cancer status was based on self-reported diagnosis. Additionally, liver function biomarkers—aspartate transaminase (AST), gamma-glutamyl transferase (GGT), and alanine transaminase (ALT)—were also included to account for potential metabolic confounders. This comprehensive adjustment aimed to minimize residual confounding and improve analytical validity.

### 2.6. Statistical analysis

All the statistical analyses accounted for the complex survey design, utilizing sample weights, stratification, and clustering adjustments. The baseline characteristics of hypertensive adults were stratified by mortality status. Continuous variables are reported as mean (SD), while categorical variables are reported as frequency (percentage). Group comparisons for continuous variables were performed using independent sample t tests (for normally distributed data) or Mann–Whitney *U* tests (for nonnormally distributed data), and for categorical variables, the Rao–Scott χ^2^ test was employed.

In this study, the scores for the six dietary patterns were categorized into four groups, and a stacked plot was used to display the distribution and trends of these dietary indicators across years (2005–2018). A survey-weighted linear regression model was subsequently used to assess the time trends of six validated dietary indicators (HEI-2020, AHEI, DASH, DII, MED, and MEDI) in the NHANES data. The study employed a correlation matrix analysis to evaluate the interrelationships between different dietary indices, using Spearman correlation coefficients to calculate the linear associations of dietary pattern scores, with a standardized presentation of the correlation matrix showing relationships ranging from −1 to +1. Additionally, a weighted multivariable Cox proportional hazards model was used to assess the associations between the six dietary indices and all-cause mortality and cardiovascular mortality. This model included two adjustment levels: Model 1 (unadjusted model) and Model 2 (adjusted for potential confounders such as sex, age, race, education level, PIR, smoking status, BMI, waist circumference, hypertension, diabetes, CKD, and cancer history). The restricted cubic spline (3 knots) method was also used to explore potential nonlinear trends between dietary indices and mortality risk. To assess the synergistic effects of dietary components, weighted quantile regression (WQS), which is an effective method for handling the impact of multiple components on outcome variables, was applied. Finally, analyses stratified by age, sex, race, and comorbidities (e.g. hypertension, diabetes) were conducted to further validate whether the relationships between dietary indices and mortality risk varied across different subgroups.

All analyses were performed using the Free Statistical analysis platform (Version 1.9, Beijing, China) [18,19]. The criterion for statistical significance was a two-tailed *p* value of less than 0.05.

### 2.7. Sensitivity analyses

To validate our primary findings, we conducted nine rigorous sensitivity analyses to reassess the association between diet and mortality: (1) propensity score matching (PSM) to balance baseline characteristics; (2) exclusion of participants with baseline malignancy or cardiovascular disease; (3) removal of deaths occurring within the first 3 follow-up years; (4) exclusion of non-Hispanic Black participants to address racial mortality disparities; (5) elimination of implausible energy intakes (<600/>3500 kcal/day for women, <800/>4200 kcal/day for men); (6) exclusion of participants with only one dietary recall; (7) removal of total energy intake from model covariates; (8) multiple imputation for missing data; and (9) k-nearest neighbors (KNN) imputation for missing values. All sensitivity analyses maintained the original weighting and adjustment strategies.

## 3. Results

### 3.1. Baseline population characteristics

A total of 13,230 hypertensive adults from the 2005–2018 NHANES dataset were included in this study, with 10,810 participants surviving, 2,420 (18.3%) dying from all-cause mortality, and 637 (4.8%) dying from cardiovascular diseases. The average age of the participants was 57.67 years, with the majority being female (51.31%). Table 1 (stratified by all-cause mortality) and Table S1 (stratified by cardiovascular mortality) present the baseline characteristics of hypertensive adults. Compared with survivors, participants who died from all-cause or cardiovascular mortality were older; were more likely to be non-Hispanic White; were more likely to live in poverty; were more likely to be former or current smokers; had lower education levels; were more likely to be widowed or divorced; and had higher prevalence rates of diabetes, cardiovascular disease, and chronic kidney disease (all comparisons *p* < 0.0001). In terms of diet, individuals who died from cardiovascular diseases had significantly lower energy intake and exhibited differences in dietary patterns (DII, HEI-2020, MEDI).

**Table 1.** Baseline characteristics of adults with hypertension in NHANES 2005–2018 stratified by all-cause mortality.

Variables	Total (*n* = 13230)	All-cause mortality		*p* value
		No (*n* = 10810)	Yes (*n* = 2420)	
Sex (%)				<0.0001
Male	6478.00 (48.69)	5161.00 (48.53)	1317.00 (49.62)	
Female	6752.00 (51.31)	5649.00 (51.47)	1103.00 (50.38)	
Age, years (mean (SD))	57.665 (14.789)	55.702 (14.288)	69.470 (11.992)	<0.0001
Race (%)				<0.0001
Mexican American	1556.00 (5.41)	1381.00 (5.80)	175.00 (3.05)	
Non-Hispanic White	5998.00 (71.05)	4509.00 (69.63)	1489.00 (79.62)	
Non-Hispanic Black	3568.00 (13.67)	3006.00 (13.91)	562.00 (12.22)	
Other race	2108.00 (9.88)	1914.00 (10.67)	194.00 (5.11)	
Family poverty–income ratio				<0.0001
<1.3	3764.00 (18.98)	2956.00 (17.79)	808.00 (26.15)	
1.3–3.5	5934.00 (42.28)	4743.00 (40.94)	1191.00 (50.31)	
>3.5	3532.00 (38.75)	3111.00 (41.27)	421.00 (23.54)	
Smoking status (%)				<0.0001
Never	6649.00 (49.82)	5683.00 (51.51)	966.00 (39.63)	
Past	4156.00 (32.14)	3152.00 (30.75)	1004.00 (40.47)	
Current	2425.00 (18.05)	1975.00 (17.74)	450.00 (19.90)	
Educational level (%)				<0.0001
High school or less	6767.00 (42.75)	5243.00 (40.24)	1524.00 (57.83)	
College or above	6463.00 (57.25)	5567.00 (59.76)	896.00 (42.17)	
Marital status (%)				
Married	7051.00 (59.08)	5939.00 (60.74)	1112.00 (49.05)	<0.0001
Widowed	1820.00 (10.72)	1126.00 (8.02)	694.00 (26.97)	
Divorced	1792.00 (12.59)	1482.00 (12.64)	310.00 (12.28)	
Separated	468.00 (2.51)	390.00 (2.56)	78.00 (2.20)	
Never married	1400.00 (9.40)	1246.00 (9.94)	154.00 (6.13)	
Living with partner	699.00 (5.70)	627.00 (6.09)	72.00 (3.37)	
BMI, kg/m^2^ (mean (SD))	31.169 (7.269)	31.393 (7.213)	29.817 (7.456)	<0.0001
Waist circumference, cm (mean (SD))	105.712 (16.006)	105.870 (16.019)	104.759 (15.895)	0.0269
Total energy intake, kcal/d (mean (SD))	1044.656 (478.720)	1064.912 (481.632)	922.817 (441.736)	<0.0001
GGT, µ/l (mean (SD))	34.102 (51.628)	32.894 (42.976)	41.365 (86.774)	0.005
ALT, µ/l (mean (SD))	26.323 (20.506)	26.663 (17.313)	24.281 (33.798)	0.0036
ATS, µ/l (mean (SD))	26.449 (17.380)	26.108 (15.816)	28.494 (24.692)	0.0035
Diabetes (%)				<0.0001
No	8632.00 (70.93)	7326.00 (73.32)	1306.00 (56.59)	<0.0001
Yes	4598.00 (29.07)	3484.00 (26.68)	1114.00 (43.41)	
CVD (%)				<0.0001
No	10494.00 (82.59)	9035.00 (85.99)	1459.00 (62.13)	<0.0001
Yes	2736.00 (17.41)	1775.00 (14.01)	961.00 (37.87)	
CKD (%)				<0.0001
No	2352.00 (15.27)	1399.00 (11.51)	953.00 (37.91)	<0.0001
Yes	10878.00 (84.73)	9411.00 (88.49)	1467.00 (62.09)	
Hyperlipidemia (%)				0.1651
No	4081.00 (29.10)	3306.00 (28.87)	775.00 (30.47)	
Yes	9149.00 (70.90)	7504.00 (71.13)	1645.00 (69.53)	
Cancer (%)				<0.0001
No	11236.00 (83.96)	9446.00 (85.84)	1790.00 (72.60)	<0.0001
Yes	1994.00 (16.04)	1364.00 (14.16)	630.00 (27.40)	
AHEI (mean (SD))	38.956 (11.195)	39.010 (11.312)	38.630 (10.464)	0.2817
DASH (Mean (SD)	22.605 (4.930)	22.543 (4.962)	22.972 (4.715)	0.0081
DII (mean (SD))	1.099 (1.675)	1.056 (1.672)	1.362 (1.666)	<0.0001
HEI-2020 (mean (SD))	51.699 (11.814)	51.566 (11.848)	52.496 (11.580)	0.0153
MED (mean (SD))	3.463 (1.338)	3.469 (1.344)	3.428 (1.307)	0.3273
MEDI (mean (SD))	3.569 (1.016)	3.579 (1.034)	3.507 (0.895)	0.0141

GGT: Gamma glutamyl transferase; ALT: alanine aminotransferase; AST: aspartate aminotransferase; CVD: cardiovascular disease; CKD: chronic kidney Disease; AHEI: Alternate Healthy Eating Index; DASH: dietary approaches to stop hypertension index; DII: Dietary Inflammatory Index; HEI-2020: Healthy Eating Index-2020; MED: Mediterranean Diet; MEDI: Mediterranean diet index in serving sizes from the PREDIMED trial.

### 3.2. Trends and correlations of the six dietary indices

**Figure 1.**
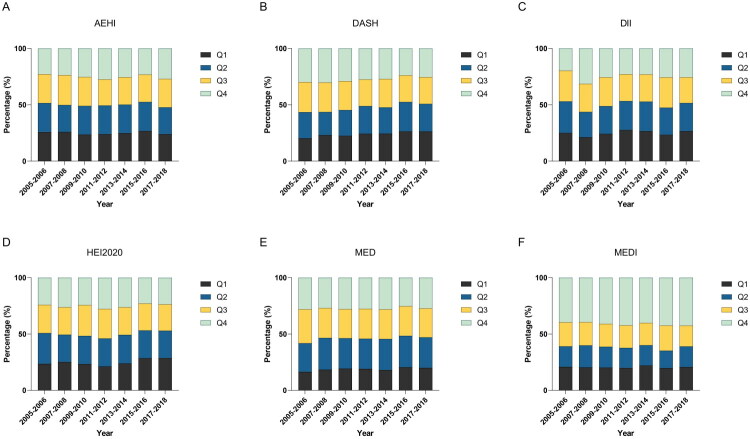
Temporal trends in quartile distributions of six dietary patterns among U.S. adults, NHANES 2005–2018.

The stacked plot (Figure 1) clearly illustrates the temporal changes in the distributions of the dietary indices from 2005 to 2018. Notably, adherence to the DASH and Mediterranean diets displayed distinct fluctuations. For DASH, the proportion of participants in the highest quartile (Q4) significantly declined over time, whereas the proportion in the lowest quartile (Q1) slightly increased. In contrast, the Mediterranean diet (MED) showed a more stable trend, with little change in the quartile distribution. AHEI, DII, HEI-2020, and MEDI exhibited relatively stable distributions over the years, with no significant changes in quartile proportions.

**Figure 2.**
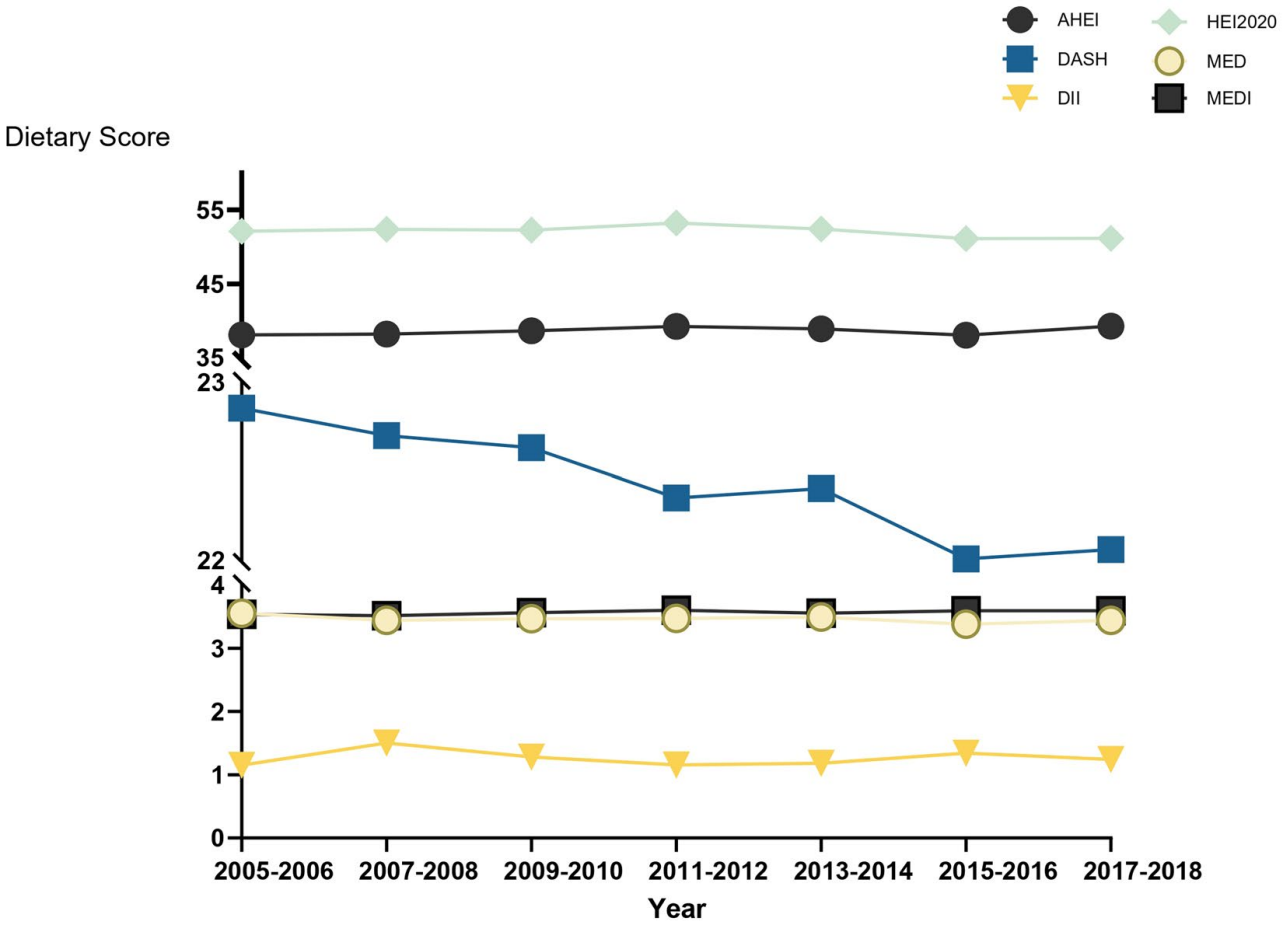
Trends in dietary scores over 7 NHANES cycles (2005–2018) in hypertensive adults.

Figure 2 displays the linear trend analysis from 2005 to 2006 to 2017–2018. Although the absolute changes were small, the DASH scores showed a statistically significant decreasing trend (*p* = 0.0005). In contrast, the MEDI scores exhibited a moderate but statistically significant increasing trend (*p* = 0.046). No significant trends were observed for the AHEI, DII, HEI-2020, or MED scores (all *p* > 0.05). The continued decline in DASH scores and the slight increase in MEDI scores suggest subtle shifts in dietary patterns over the study period, although overall changes were still modest.

**Figure 3.**
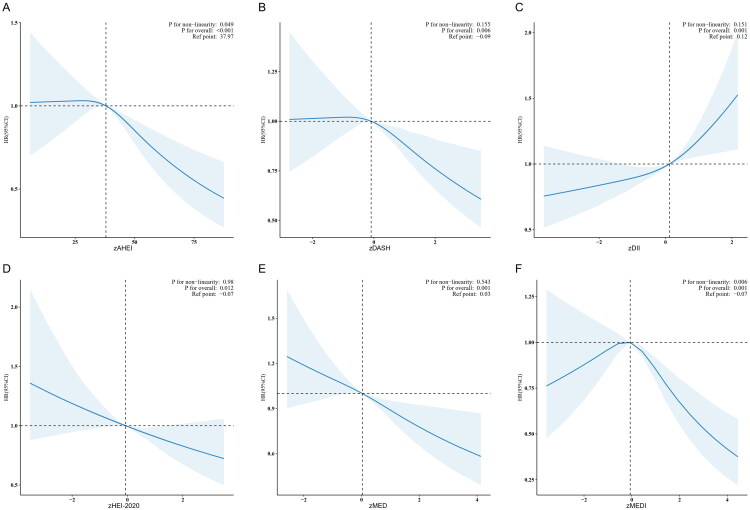
Restricted cubic spline plot of the association between dietary patterns and all-cause mortality. Solid and dashed lines represent the predicted value and 95% confidence intervals. The models were adjusted for sex, age, race, educational level, family poverty–income ratio, marital status, smoking status, BMI, waist circumference, GGT, AST, ALT, total energy intake, diabetes, CVD, CKD, hyperlipidemia, and cancer.

Spearman correlation analysis revealed strong positive correlations between the HEI-2020 and DASH (*r* = 0.83) and moderate positive correlations between MED and AHEI (*r* = 0.80), DASH (*r* = 0.78), and HEI-2020 (*r* = 0.74), as well as between HEI-2020 and DASH (*r* = 0.78) and AHEI (*r* = 0.74). The correlation between MEDI and other dietary patterns was weaker, and DII was generally negatively correlated with the other dietary indices, especially with MED (*r* = −0.53), HEI-2020 (*r* = −0.50), and DASH (*r* = −0.49) (Figure S2). These temporal and correlational patterns lay the groundwork for exploring how adherence to different dietary patterns is related to mortality outcomes.

### 3.3. Dietary patterns and all-cause and CVD mortality

A series of weighted Cox proportional hazards regression analyses revealed significant associations between the six dietary indices and all-cause mortality outcomes, regardless of whether the dietary indices were treated as continuous or categorical variables. As continuous variables, several dietary indices were significantly negatively associated with all-cause mortality risk: zAHEI (HR = 0.90, 95% CI: 0.85–0.96, *p* < 0.001), zDASH (HR = 0.92, 95% CI: 0.87–0.97, *p* = 0.005), zHEI-2020 (HR = 0.91, 95% CI: 0.86–0.97, *p* = 0.003), zMED (HR = 0.90, 95% CI: 0.85–0.95, *p* < 0.001), and zMEDI (HR = 0.93, 95% CI: 0.89–0.98, *p* = 0.008). In contrast, the zDII was positively correlated with all-cause mortality (HR = 1.14, 95% CI: 1.06–1.22, *p* < 0.001) (Table 2).

**Table 2.** Hazard ratios of mortality according to different dietary indices among hypertensive adults.

Variable	All-cause mortality				Cardiovascular mortality			
	Model 1		Model 2		Model 1		Model 2	
	HR (95% CI)	*p* value	HR (95% CI)	*p* value	HR (95% CI)	*p* value	HR (95% CI)	*p* value
zAHEI								
Continuous	0.97 (0.92, 1.02)	0.261	0.9 (0.85, 0.96)	<0.001	0.95 (0.86, 1.05)	0.345	0.9 (0.80, 1.01)	0.075
Quartile								
Q1	1 (Ref)		1 (Ref)		1 (Ref)		1 (Ref)	
Q2	1.24 (1.07, 1.43)	0.005	1.03 (0.88, 1.20)	0.751	1.15 (0.88, 1.51)	0.302	0.96 (0.73, 1.26)	0.771
Q3	1.09 (0.93, 1.27)	0.274	0.89 (0.78, 1.01)	0.062	0.97 (0.73, 1.28)	0.819	0.8 (0.61, 1.05)	0.105
Q4	0.98 (0.83, 1.15)	0.768	0.8 (0.68, 0.93)	0.005	0.97 (0.71, 1.32)	0.827	0.83 (0.59, 1.16)	0.279
*p* value for trend		0.402		0.001		0.552		0.176
zDASH								
Continuous	1.04 (0.98, 1.10)	0.186	0.92 (0.87, 0.97)	0.005	0.93 (0.89 ∼ 0.96)	<0.001	0.92 (0.88 ∼ 0.96)	<0.001
Quartile								
Q1	1 (Ref)		1 (Ref)		1 (Ref)		1 (Ref)	
Q2	1.28 (1.07, 1.52)	0.006	1 (0.87, 1.160)	0.976	1.43 (1.05, 1.95)	0.023	1.08 (0.80, 1.46)	0.609
Q3	1.33 (1.11, 1.60)	0.002	0.97 (0.81, 1.16)	0.738	1.26 (0.95, 1.68)	0.113	0.92 (0.69, 1.22)	0.544
Q4	1.22 (1.02, 1.46)	0.031	0.83 (0.70, 0.98)	0.03	1.41 (1.05, 1.89)	0.021	0.94 (0.69, 1.29)	0.718
*p* value for trend		0.056		0.019		0.073		0.465
zDII								
Continuous	1.21 (1.14, 1.28)	<0.001	1.14 (1.06, 1.22)	<0.001	1.19 (1.07, 1.33)	0.001	1.1 (0.96, 1.25)	0.183
Quartile								
Q1	1 (Ref)		1 (Ref)		1 (Ref)		1 (Ref)	
Q2	1.16 (0.98, 1.38)	0.089	1.1 (0.93, 1.31)	0.255	1.22 (0.91, 1.65)	0.185	1.13 (0.85, 1.51)	0.382
Q3	1.28 (1.09, 1.51)	0.002	1.13 (0.95, 1.35)	0.176	1.32 (0.98, 1.76)	0.064	1.12 (0.79, 1.60)	0.519
Q4	1.63 (1.40, 1.91)	<0.001	1.35 (1.10, 1.65)	0.004	1.64 (1.24, 2.16)	<0.001	1.29 (0.91, 1.82)	0.159
*p* value for trend		<0.001		0.004		<0.001		0.208
zHEI-2020								
Continuous	1.05 (0.99, 1.11)	0.085	0.91 (0.86, 0.97)	0.003	1.11 (0.99, 1.25)	0.069	0.97 (0.86, 1.10)	0.672
Quartile								
Q1	1 (Ref)		1 (Ref)		1 (Ref)		1 (Ref)	
Q2	1.17 (1.03, 1.33)	0.013	0.99 (0.86, 1.15)	0.933	1.17 (0.89, 1.54)	0.250	0.97 (0.72, 1.31)	0.859
Q3	1.26 (1.09, 1.45)	0.001	0.96 (0.83, 1.12)	0.634	1.3 (0.99, 1.71)	0.062	0.97 (0.73, 1.28)	0.807
Q4	1.17 (0.99, 1.38)	0.058	0.8 (0.68, 0.95)	0.011	1.29 (0.95, 1.76)	0.108	0.89 (0.65, 1.22)	0.482
*p* value for trend		0.036		0.005		0.096		0.506
Zmed								
Continuous	0.94 (0.89, 1.00)	0.049	0.9 (0.85, 0.95)	<0.001	0.96 (0.87, 1.07)	0.475	0.92 (0.82, 1.03)	0.155
Quartile								
Q1	1 (Ref)		1 (Ref)		1 (Ref)		1 (Ref)	
Q2	0.92 (0.78, 1.08)	0.287	0.87 (0.74, 1.01)	0.068	0.91 (0.68, 1.23)	0.553	0.85 (0.63, 1.15)	0.3
Q3	0.92 (0.78, 1.08)	0.297	0.84 (0.73, 0.96)	0.013	0.91 (0.66, 1.25)	0.562	0.8 (0.58, 1.10)	0.165
Q4	0.84 (0.71, 1.00)	0.052	0.74 (0.62, 0.87)	<0.001	0.85 (0.62, 1.17)	0.312	0.74 (0.52, 1.04)	0.086
*p* value for trend		0.07		<0.001		0.33		0.08
zMEDI								
Continuous	0.94 (0.90, 0.99)	0.01	0.93 (0.89, 0.98)	0.008	0.91 (0.83, 1.00)	0.053	0.92 (0.83, 1.03)	0.151
Quartile								
Q1	1 (Ref)		1 (Ref)		1 (Ref)		1 (Ref)	
Q2	1.47 (1.23, 1.75)	<0.001	1.11 (0.94, 1.30)	0.222	1.46 (1.06, 2.01)	0.02	1.03 (0.76, 1.40)	0.844
Q3	1.4 (1.18, 1.65)	<0.001	1.02 (0.88, 1.18)	0.77	1.27 (0.90, 1.81)	0.179	0.9 (0.64, 1.26)	0.541
Q4	1.07 (0.92, 1.24)	0.401	0.95 (0.83, 1.10)	0.527	1.01 (0.75, 1.37)	0.942	0.94 (0.69, 1.27)	0.671
*p* value for trend		0.631		0.212		0.47		0.503

HR = hazard ratio; CI = confidence interval. Model 1 was unadjusted; Model 2 was adjusted for sex, age, race, educational level, family poverty–income ratio, marital status, smoking status, BMI, waist circumference, GGT, AST, ALT, total energy intake, diabetes, CVD, CKD, hyperlipidemia, and cancer.

When dietary indices were categorized into quartiles, multiple dietary indices were associated with significantly lower all-cause mortality risk in the highest quartile (Q4) compared to the lowest quartile (Q1): zAHEI (HR = 0.80, 95% CI: 0.68–0.93, *p* = 0.005), zDASH (HR = 0.83, 95% CI: 0.70–0.98, *p* = 0.03), and zMED (HR = 0.74, 95% CI: 0.62–0.87, *p* < 0.001). On the other hand, the zDII revealed a significantly greater mortality risk in the highest quartile (Q4) (HR = 1.35, 95% CI: 1.10–1.65, *p* = 0.004). However, no significant associations or trends were observed between zHEI-2020 and zMEDI across quartiles (*p* > 0.05).

For cardiovascular mortality, only zDASH showed a significant association (HR = 0.92, 95% CI: 0.88–0.96, *p* < 0.001), whereas no significant associations with cardiovascular mortality were found for other dietary patterns.

On the basis of restricted cubic splines, the association between dietary patterns and all-cause mortality followed a dose–response pattern, whereas no such association was observed with cardiovascular mortality (Figure S3).

### 3.4. Subgroup and sensitivity analyses

To further evaluate the consistency and generalizability of the observed associations, we conducted subgroup and sensitivity analyses. Apart from an interaction between MEDI and mortality risk in the diabetes group, no significant interactions were detected between dietary patterns and mortality risk across other subgroups (all interaction *p* values >0.05) (Figures S4–S7). A series of sensitivity analyses were also performed to assess the robustness of the primary results regarding dietary patterns and mortality risk, which yielded similar findings (Tables S2–S10).

### 3.5. Combined effects of dietary patterns on all-cause and cardiovascular deaths

Table S11 shows that the DII was significantly associated with all-cause mortality (HR = 1.237, 95% CI: 1.134–1.351, *p* < 0.001), whereas the HEI-2020 and MEDI had protective effects against all-cause mortality (HEI-2020: HR = 0.929, 95% CI: 0.893–0.966, *p* < 0.001; MEDI: HR = 0.513, 95% CI: 0.327–0.804, *p* = 0.003). Both DASH and HEI-2020 were associated with reduced cardiovascular mortality (HR = 0.831, 95% CI: 0.711–0.971, *p* = 0.0195; HR = 0.883, 95% CI: 0.797–0.979, *p* = 0.0171).

Figures S8 and S9 present the contribution weights of dietary components in the WQS–mortality models. For all-cause mortality, in the DII model, n-3 fatty acids (14.29%) and n-6 fatty acids (5.65%) were the major contributing components and showed inverse associations with mortality, indicating protective roles. Caffeine (8.18%) also contributed notably, but its directionality was less consistent. In the HEI-2020 model, dairy accounted for the greatest contribution (49.48%) and was associated with lower mortality risk, suggesting a protective effect. Sodium (25.69%) was positively associated with mortality, which aligns with its known adverse cardiovascular impact, whereas total fruits (8.78%) were protective. In the MEDI model, free fats and oils contributed the most (30.81%) and were positively associated with all-cause mortality, indicating a potentially harmful effect when consumed in excess. Alcohol (10%) and sugary beverages (8.81%) were also positively associated.

For cardiovascular mortality, the DII model identified low-fat dairy (30.3%) and whole grains (20.5%) as protective contributors, whereas sugary beverages (15.94%) were positively associated with increased risk. In the HEI-2020 model, dairy (20.64%) and whole grains (16.3%) were again associated with reduced mortality, whereas added sugars (18.65%) had a harmful effect. Overall, dairy products and whole grains were consistently associated with reduced mortality risk across models, whereas sodium, added sugars, sugary beverages, and free fats/oils tended to exhibit harmful associations.

## 4. Discussion

This study systematically evaluated six widely used dietary pattern indices and their associations with all-cause and cardiovascular mortality in hypertensive adults using nationally representative NHANES data. Our findings demonstrated that greater adherence to healthful dietary patterns—specifically AHEI, DASH, HEI-2020, MED, and MEDI—was significantly associated with reduced risk of all-cause mortality. In contrast, a higher DII score, indicative of a proinflammatory diet, was associated with increased mortality risk. Among these indices, only the DASH score exhibited a robust and independent inverse association with cardiovascular mortality, suggesting its particular cardioprotective relevance for hypertensive populations. These results reinforce the vital role of dietary quality in modulating health outcomes and provide new comparative evidence with respect to the predictive power of different dietary scoring systems.

In addition to mortality associations, we also observed modest but significant changes in adherence to dietary patterns over time. Specifically, DASH scores showed a declining trend between 2005 and 2018, whereas MEDI scores increased slightly. These shifts may reflect broader changes in the U.S. food environment, sociocultural attitudes toward nutrition, and accessibility to different dietary components. For example, the decreasing adherence to the DASH diet could be attributed to increased consumption of processed and high-sodium foods, fueled by the prevalence of fast food and limited nutritional literacy in certain populations [20]. Conversely, the gradual rise in MEDI scores may correspond to growing public interest in anti-inflammatory, plant-based, or Mediterranean-style diets, driven by media coverage and evolving dietary guidelines [21–24]. Understanding these temporal patterns is critical, as they may influence the population-level effectiveness of dietary interventions and public health strategies targeting hypertensive individuals.

In this study, we further analyzed the associations between various healthy dietary patterns and all-cause mortality in hypertensive patients and explored heterogeneity across subgroups. AHEI, DASH, and HEI-2020 are widely researched and used dietary pattern scoring tools. The AHEI particularly emphasizes the intake of vegetables, fruits, whole grains, nuts, fish, and healthy fatty acids while encouraging the reduction of trans fats and processed meats. Numerous studies have shown that higher AHEI scores are closely related to reductions in cardiovascular disease and mortality risk [25–27]. Similarly, DASH, by reducing sodium intake and increasing potassium and calcium, effectively lowers blood pressure and is closely linked to reduced cardiovascular event risk [28]. This dietary pattern not only helps control hypertension but has also been shown to reduce all-cause mortality [29–31]. Moreover, the HEI-2020 comprehensively assesses diet quality, integrating nutrients and food quality to evaluate the relationship between diet and health in a more holistic manner. Previous studies have also demonstrated that dietary association with good HEI scores can lower all-cause mortality [31–34]. A recent NHANES-based cohort analysis (2005–2018) revealed that participants in the highest adherence tertile for AHEI, DASH, HEI-2020, and MED had 27–41% lower all-cause mortality than did those in the lowest tertile (HR range: 0.59–0.73; all *p* < 0.05) [35]. These dietary patterns may exert protective effects through multiple biological pathways, particularly reducing systemic low-grade inflammation (e.g. lowering CRP and IL-6), improving endothelial function, and ensuring increased intake of blood pressure-relevant micronutrients (potassium, magnesium, calcium, and polyphenolic compounds) [36,37,7].

We also included MED and MEDI, with MED emphasizing plant-based foods, fish, olive oil, and moderate alcohol intake, while MEDI quantifies adherence to the Mediterranean diet to assess corresponding health benefits [26,31]. A 25-year follow-up study from the Women’s Health Study revealed a 23% reduction in all-cause mortality among participants who strongly adhered to the Mediterranean diet [38]. Additionally, another study based on the NHANES database revealed that higher Mediterranean diet scores were significantly associated with reduced all-cause mortality in patients with coronary artery disease or a history of stroke [39]. In addition to these epidemiological associations, mechanistic studies provide further support for the beneficial effects of Mediterranean-style dietary patterns. A 2025 systematic review and meta-analysis of 16 randomized clinical trials demonstrated that a Mediterranean diet enriched with olive oil significantly reduced the levels of systemic inflammatory markers (CRP, IL-6, TNF-α, ICAM-1, and VCAM-1) and improved endothelial function *via* increased flow-mediated dilation (FMD) [36]. Additionally, a randomized controlled feeding trial in high cardiometabolic risk individuals revealed that adherence to the Mediterranean diet improved blood pressure, vascular reactivity, and lipid profiles after eight weeks of intervention [37]. These findings suggest that the protective effects of MED and MEDI may be mediated through improved diet quality, suppression of low-grade systemic inflammation, and enhanced vascular health—mechanisms that are especially relevant to hypertensive populations—as supported by our subgroup analyses.

The positive impacts of these dietary patterns are achieved through mechanisms such as improved diet quality, enhanced vascular health, and reduced inflammation, which, as our study confirmed, can lower all-cause mortality in hypertensive patients.

In contrast to health-promoting dietary patterns, diets with higher DII scores exhibit the opposite trend. Higher DII scores typically reflect diets rich in processed meats, refined sugars, red meats, and high-sodium foods, which are prone to induce chronic low-grade inflammation. Such inflammation is closely linked to multiple chronic diseases, including cardiovascular disease, diabetes, and cancer [40]. Previous studies have also shown that individuals with higher inflammation levels generally have greater cardiovascular disease and mortality risks [41,42], which is consistent with our findings. Notably, a recent NHANES-based cohort study in hypertensive adults demonstrated that those in the highest DII quartile had approximately 35% higher cardiovascular mortality (especially among women) and a significant increase in non-CV mortality, revealing nonlinear dose–response patterns [43]. Furthermore, our WQS regression analysis highlighted that specific dietary components—such as lower intake of unsaturated fatty acids—made disproportionately large contributions to mortality risk within the DII model. This finding is consistent with mechanistic studies showing that unsaturated fatty acids, polyphenols, and micronutrients reduce inflammation and improve metabolic function [44–47], thereby helping lower mortality risk. Therefore, we suggest that hypertensive patients reduce their intake of proinflammatory foods—such as processed meats, refined carbohydrates, and high-sodium products—to mitigate inflammation and improve long-term health outcomes.

An unexpected finding in our study was that, in addition to the DASH diet, no other dietary patterns, including the Mediterranean diet and healthy plant-based diets, were significantly associated with cardiovascular mortality in hypertensive patients. This finding deviates from our initial hypothesis, and even after conducting a series of sensitivity analyses, the results remained consistent. Reviewing past studies, we can find several possible explanations. For example, an 18-year follow-up study of 11,376 men and women without a history of myocardial infarction or stroke revealed no association between a Mediterranean diet (MED) and cardiovascular mortality [48]. Similarly, another study on hypertensive patients failed to confirm a significant association between the Healthy Eating Index 2015 (HEI-2015) and cardiovascular disease-specific mortality in hypertensive patients [26]. These findings suggest that although some dietary patterns show cardiovascular health benefits in the general population, their association with cardiovascular mortality in hypertensive patients may not be significant [49]. This may be because the benefits of these dietary patterns are more evident in the prevention of cardiovascular disease incidence rather than directly lowering cardiovascular mortality in those already diagnosed with hypertension. The pathophysiology of hypertension is complex and involves not only blood pressure control but also inflammation, metabolic disturbances, and endothelial dysfunction [50]. Some dietary patterns may have a positive effect on blood pressure control but have limited impact on other cardiovascular risk factors, thus failing to significantly lower cardiovascular mortality. In contrast, only DASH exhibited a robust and independent inverse association with cardiovascular mortality. This finding supports emerging evidence from the DASH4D randomized trial in type 2 diabetic patients, where a sodium‑restricted DASH diet lowered systolic blood pressure by ∼4.6 mm Hg [8]. The combination of DASH with time‑restricted eating also led to increased BP reduction in primary hypertension patients in a recent RCT [51], which may help explain the unique cardioprotective effects of DASH in hypertensive populations.

Our WQS regression analysis identified sugary beverages and low-fat dairy as significant dietary contributors to cardiovascular mortality in hypertensive individuals. Sugary beverages, particularly sugar-sweetened drinks, are well known to impair glycemic control and exacerbate lipid metabolism, which increases cardiovascular risk  [52–54]. A recent umbrella review of 47 meta-analyses involving over 22 million individuals classified the evidence linking sugar-sweetened beverage consumption to cardiovascular disease as ‘convincing’, noting a dose–response association with an approximately 10–30% greater risk of coronary heart disease and hypertension per daily serving [55].

Conversely, low-fat dairy products—rich in calcium, potassium, magnesium, and high-quality protein—have been inversely associated with hypertension and CVD risk. A 2025 meta-analysis in Nature Communications revealed that each additional daily serving of low-fat dairy was linked to a 4% lower stroke risk and modest reduction in cardiovascular outcomes [56]. Furthermore, a Japanese cross-sectional study revealed that increased dairy intake was associated with modest reductions in systolic blood pressure (β ≈ −0.021 per serving; *p* ≈ 0.044), possibly *via* the modulation of phosphorus metabolism and IL-6 [57]. These findings align with our WQS regression model, where low intake of protective nutrients in low-fat dairy is correlated with increased cardiovascular mortality. Collectively, the evidence suggests that hypertensive patients benefit from limiting sugary drinks while ensuring adequate intake of nutrient-dense low-fat dairy to regulate glucose/lipid metabolism, reduce inflammation, and control blood pressure.

Despite the strengths of this study—namely, the large, nationally representative NHANES sample and the use of multiple validated dietary scoring systems—several limitations must be acknowledged, each accompanied by suggested future improvements. First, the observational nature of the study limits causal inference, even though Cox proportional hazards models and sensitivity analyses were applied. Residual confounding due to unmeasured factors such as physical activity, medication adherence, and psychosocial stress cannot be entirely excluded. To address this, future research should employ randomized controlled trials (RCTs) or well-designed prospective cohort studies with repeated dietary assessments to enhance causal inference. Second, dietary intake was based on self-reported 24-h dietary recalls, which are susceptible to recall bias and daily variation and may not accurately capture long-term dietary behavior. In response, future studies should consider incorporating multiple dietary assessments or objective nutritional biomarkers to improve the accuracy of exposure measurements. Third, while a broad range of covariates was included, NHANES lacks information regarding key biological modulators such as gut microbiota profiles, genetic susceptibility, and inflammatory biomarkers, which limits mechanistic understanding. To overcome this, future investigations should integrate metabolomic, microbiome, and immunologic data to elucidate the biological pathways linking diet to mortality risk. Fourth, although several dietary indices were evaluated, important indices—such as plant-based or low-carbohydrate diet scores—were not included. Furthermore, existing scoring systems may require updates as dietary guidelines and food environments evolve. Accordingly, future work should expand the scope of dietary indices and adapt existing metrics in response to changing nutritional standards. Fifth, the relatively small number of cardiovascular deaths (637 out of 13,230 participants) may reduce the statistical power to detect significant associations between certain dietary indices and cardiovascular mortality. This limitation highlights the need for future studies with larger sample sizes or specific populations to further investigate these associations. Moreover, although the association between HEI-2020 and cardiovascular mortality (HR = 0.883) reached statistical significance—indicating an approximate 12% relative risk reduction—this study’s reliance on cross-sectional dietary recall and observational follow-up limits us to reporting relative measures only, which cannot be directly translated into clinically meaningful absolute risk differences or intervention benefits. Future investigations, ideally employing more rigorous designs (e.g. randomized controlled trials), should incorporate absolute risk reduction (ARR) and number needed to treat (NNT) metrics to more accurately quantify the true clinical impact of dietary interventions. Finally, a limitation of this study is the overlap among dietary indices, such as HEI-2020, DASH, AHEI, and MED, which share common components (e.g. whole grains, fruits, vegetables). This overlap may lead to multicollinearity, making it difficult to distinguish the independent effects of each dietary pattern on health outcomes. Future studies could consider applying alternative statistical approaches, such as stepwise regression or ridge regression, to better address multicollinearity and identify the unique contributions of each dietary pattern [58,59].

## 5. Conclusion

These findings highlight the critical role of overall dietary quality in shaping long-term health outcomes among hypertensive individuals. These findings provide robust evidence supporting the integration of dietary assessment tools into hypertension management strategies and underscore the need for public health efforts to promote sustainable, anti-inflammatory, and nutrient-rich eating patterns. Furthermore, our results suggest that dietary interventions should be tailored on the basis of individual risk profiles and that continued refinement of dietary scoring systems is necessary to capture evolving nutritional environments. Ultimately, this study offers meaningful insights for clinicians, researchers, and policy-makers aiming to reduce mortality risk through precision nutrition and evidence-based dietary guidance.

## Supplementary Material

Supplemental Material

## Data Availability

The datasets used and analyzed during the current study are available in the NHANES (https://wwwn.cdc.gov/nchs/nhanes/Default.aspx). The datasets generated for this study are available upon request to the corresponding author.
