# Supplementary material for: Associations of dietary indices with risk of all-cause and cardiovascular mortality in hypertensive adults
Source: Ann Med. 2025 Nov 15;57(1):2584427. doi: 10.1080/07853890.2025.2584427 (PMC12621336; doi:10.1080/07853890.2025.2584427)
Supplement: Supplemental Material [file IANN_A_2584427_SM3071.zip › suppl_data/Supplemental Methods.docx]

Supplemental Methods

### **2.3.1. Alternative Healthy Eating Index (AHEI)**

### The Alternative Healthy Eating Index (AHEI) is a validated dietary scoring system designed to predict chronic disease risk, with particular emphasis on cardiovascular health. Developed as an enhancement to the original Healthy Eating Index (HEI), the AHEI incorporates 11 components: six adequacy components (vegetables, fruits, whole grains, nuts/legumes, long-chain omega-3 fats, and polyunsaturated fatty acids) and five moderation components (sugar-sweetened beverages/fruit juice, red/processed meat, trans fat, sodium, and alcohol). Each component is scored from 0 (worst) to 10 (best), with total scores ranging from 0 to 110. Higher AHEI scores indicate greater adherence to dietary patterns associated with lower morbidity and mortality(1).

### **2.3.2. Dietary Approaches to Stop Hypertension (DASH)**

The DASH diet emphasizes the consumption of fruits, vegetables, low-fat dairy products, whole grains, and lean meat proteins, while restricting the intake of saturated fats, cholesterol, and sodium(2). Our DASH score follows the standard eight-component system: high intake of fruits, vegetables, nuts/legumes, whole grains, and low-fat dairy products; moderate intake of lean meats, poultry, and fish; and limited intake of sodium, red/processed meats, and sugary beverages. Each component is scored based on whether the intake aligns with the principles of the DASH diet, with scores for each component ranging from 0 to 5. The total score ranges from 0 to 40, with higher scores indicating better adherence to DASH principles(3).

#### **2.3.3. Dietary Inflammatory Index (DII)**

The Dietary Inflammatory Index (DII) assesses the inflammatory potential of diet by scoring 27 food parameters (e.g., macronutrients, micronutrients, bioactive compounds) based on their established associations with inflammatory biomarkers. In the DII scoring system, pro-inflammatory components (such as saturated fats and refined carbohydrates) are assigned positive scores, while anti-inflammatory components (such as fiber and ω-3 fatty acids) are given negative scores. The total DII score reflects the net inflammatory potential of the diet: higher scores indicate a stronger pro-inflammatory potential, while lower scores indicate a stronger anti-inflammatory potential(4).

#### **2.3.4. Healthy Eating Index-2020 (HEI-2020)**

The Healthy Eating Index 2020 (HEI-2020) measures adherence to the 2020-2025 Dietary Guidelines for Americans (DGA) through 13 components. It includes nine adequacy components (total fruits, whole fruits, vegetables, legumes, whole grains, dairy, total protein foods, seafood/plant proteins, and fatty acid ratio) and four moderation components (refined grains, sodium, added sugars, and saturated fats). Each component is scored based on the degree to which the intake aligns with the recommended targets, with scores ranging from 0 to 100%, for a total score of up to 100 points. The HEI-2020 was updated to include refined standards for added sugars and fatty acid ratios, reflecting the evolving body of nutritional evidence(5, 6).

#### **2.3.5. Mediterranean Diet Score (MED)**

The Mediterranean Diet Score (MED) reflects adherence to the traditional Mediterranean diet, characterized by high consumption of plant-based foods, olive oil, fish, and moderate alcohol intake. We utilized a scoring system composed of ten components: vegetables, fruits, nuts, legumes, whole grains, fish, the ratio of monounsaturated to saturated fats (MtSR), dairy (reverse scoring), meats (reverse scoring), and alcohol (moderate intake scored positively). Each component is scored on a scale from 0 to 1, with a total score ranging from 0 to 10. Higher MED scores are associated with established heart metabolic benefits(7, 8).

#### **2.3.6. Mediterranean Dietary Index (MEDI)**

The Mediterranean Dietary Index (MEDI) is a composite scoring system used to assess adherence to the traditional Mediterranean dietary pattern. The index evaluates 11 key dietary components: high consumption of vegetables, fruits, nuts, legumes, whole grains, and fish; moderate alcohol intake; use of olive oil as the primary fat source; and the limitation of dairy products, meats, and sweets. Each component is scored based on predefined intake thresholds, with scores ranging from 0 to 1 and a total score ranging from 0 to 11. Higher MEDI scores indicate greater adherence to the Mediterranean diet(9, 10).

#### **2.3.7. Dietary Assessment Methodology**

All dietary data were derived from two non-consecutive 24-hour dietary recalls, which were collected by trained interviewers using the Automated Multiple-Pass Method (AMPM) from the U.S. Department of Agriculture. Preliminary analysis was based on the first face-to-face recall data, while the second telephone recall was used for quality control and reliability assessment. Nutrient intake was calculated using the Food and Nutrient Database for Dietary Studies (FNDDS), corresponding to each NHANES cycle version, while food group equivalents were derived from the Food Patterns Equivalents Database (FPED). To account for variations in energy intake, total energy intake for all dietary components was adjusted using the residual method.

For analytical purposes, each dietary index score was categorized into quartiles (Q1-Q4) based on the population distribution, where Q1 represents the lowest adherence and Q4 represents the highest adherence. This approach helps examine the potential dose-response relationship between dietary quality and clinical outcomes, while accounting for nonlinear associations. The scoring algorithms for AHEI, DASH, DII, HEI-2020, MED, and MEDI were implemented based on standardized protocols from their original publications, with slight adjustments made according to NHANES dietary assessment methodology. To facilitate direct comparison of the effects of different dietary indices, all scores were standardized through Z-score conversion prior to analysis. This standardization method allows for the quantitative evaluation of the relative association between different dietary patterns and health outcomes, while accounting for differences among scoring indices.

References

1. Macdiarmid JI. The food system and climate change: are plant-based diets becoming unhealthy and less environmentally sustainable? Proc Nutr Soc. 2022;81(2):162-7.

2. Appel LJ, Moore TJ, Obarzanek E, Vollmer WM, Svetkey LP, Sacks FM, et al. A clinical trial of the effects of dietary patterns on blood pressure. DASH Collaborative Research Group. N Engl J Med. 1997;336(16):1117-24.

3. Filippou CD, Tsioufis CP, Thomopoulos CG, Mihas CC, Dimitriadis KS, Sotiropoulou LI, et al. Dietary Approaches to Stop Hypertension (DASH) Diet and Blood Pressure Reduction in Adults with and without Hypertension: A Systematic Review and Meta-Analysis of Randomized Controlled Trials. Adv Nutr. 2020;11(5):1150-60.

4. Shivappa N, Steck SE, Hurley TG, Hussey JR, Hébert JR. Designing and developing a literature-derived, population-based dietary inflammatory index. Public Health Nutr. 2014;17(8):1689-96.

5. Krebs-Smith SM, Pannucci TE, Subar AF, Kirkpatrick SI, Lerman JL, Tooze JA, et al. Update of the Healthy Eating Index: HEI-2015. J Acad Nutr Diet. 2018;118(9):1591-602.

6. Shams-White MM, Pannucci TE, Lerman JL, Herrick KA, Zimmer M, Meyers Mathieu K, et al. Healthy Eating Index-2020: Review and Update Process to Reflect the Dietary Guidelines for Americans,2020-2025. J Acad Nutr Diet. 2023;123(9):1280-8.

7. deKoning L, Anand SS. Adherence to a Mediterranean diet and survival in a Greek population. Trichopoulou A, Costacou T, Bamia C, Trichopoulos D. N Engl J Med 2003; 348: 2599-608. Vasc Med. 2004;9(2):145-6.

8. O'Malley K, Willits-Smith A, Rose D. Popular diets as selected by adults in the United States show wide variation in carbon footprints and diet quality. Am J Clin Nutr. 2023;117(4):701-8.

9. Trichopoulou A, Costacou T, Bamia C, Trichopoulos D. Adherence to a Mediterranean diet and survival in a Greek population. N Engl J Med. 2003;348(26):2599-608.

10. Sofi F, Abbate R, Gensini GF, Casini A. Accruing evidence on benefits of adherence to the Mediterranean diet on health: an updated systematic review and meta-analysis. Am J Clin Nutr. 2010;92(5):1189-96.
