# Supplementary material for: Associations of dietary indices with risk of all-cause and cardiovascular mortality in hypertensive adults
Source: Ann Med. 2025 Nov 15;57(1):2584427. doi: 10.1080/07853890.2025.2584427 (PMC12621336; doi:10.1080/07853890.2025.2584427)
Supplement: Supplemental Material [file IANN_A_2584427_SM3071.zip › suppl_data/Supplementary Figure Legend.docx]

****Figure S1.** The patient selection process for this study.**

****Figure S2.** The pairwise Spearman correlation coefficient between different dietary evaluation models.**

**Figure S3.** Restricted cubic spline plot of the association between dietary patterns and cardiovascular mortality. Solid and dashed lines represent the predicted value and 95% confidence intervals. The models were adjusted for sex, age, race, educational level, family poverty-income ratio, marital status, smoking status, BMI, waist circumference, GGT, AST, ALT, total energy intake, diabetes, CVD, CKD, hyperlipidemia, and cancer.

****Figure S4.** Subgroup analysis of the association between zAHEI, zDASH, zDII, and** all-cause mortality**. Each stratification was adjusted for** sex, age, race, educational level, family poverty-income ratio, marital status, smoking status, BMI, waist circumference, GGT, AST, ALT, total energy intake, diabetes, CVD, CKD, hyperlipidemia, and cancer. **Except the stratification factor itself. Diamonds indicate OR, with horizontal lines indicating 95% CIs.**

****Figure S5.** Subgroup analysis of the association between zHEI-2020, zMED, zMEDI, and** all-cause mortality**. Each stratification was adjusted for** sex, age, race, educational level, family poverty-income ratio, marital status, smoking status, BMI, waist circumference, GGT, AST, ALT, total energy intake, diabetes, CVD, CKD, hyperlipidemia, and cancer. **Except the stratification factor itself. Diamonds indicate OR, with horizontal lines indicating 95% CIs.**

****Figure S6.** Subgroup analysis of the association between zAHEI, zDASH, zDII, and** cardiovascular mortality**. Each stratification was adjusted for** sex, age, race, educational level, family poverty-income ratio, marital status, smoking status, BMI, waist circumference, GGT, AST, ALT, total energy intake, diabetes, CVD, CKD, hyperlipidemia, and cancer. **Except the stratification factor itself. Diamonds indicate OR, with horizontal lines indicating 95% CIs.**

****Figure S7.** Subgroup analysis of the association between zHEI-2020, zMED, zMEDI, and** cardiovascular mortality**. Each stratification was adjusted for** sex, age, race, educational level, family poverty-income ratio, marital status, smoking status, BMI, waist circumference, GGT, AST, ALT, total energy intake, diabetes, CVD, CKD, hyperlipidemia, and cancer. **Except the stratification factor itself. Diamonds indicate OR, with horizontal lines indicating 95% CIs.**

**Figure S8.** Contribution weights of dietary components in the WQS-all-cause mortality model.

**Figure S9.** Contribution weights of dietary components in the WQS-cardiovascular mortality model.
