# Supplementary material for: Associations of dietary indices with risk of all-cause and cardiovascular mortality in hypertensive adults
Source: Ann Med. 2025 Nov 15;57(1):2584427. doi: 10.1080/07853890.2025.2584427 (PMC12621336; doi:10.1080/07853890.2025.2584427)
Supplement: Supplemental Material [file IANN_A_2584427_SM3071.zip › suppl_data/Table S1.docx]

**Table S1** Baseline characteristics of adults with hypertension in NHANES 2005-2018 stratified by cardiovascular mortality.

| Variables | Total (n = 13230) | Cardiovascular mortality | | *P* value |
| --- | --- | --- | --- | --- |
|  |  | No (n = 12593) | Yes (n = 637) |  |
| Sex (%) |  |  |  | 0.0011 |
| Male | 6478.00 (48.69) | 6126.00 (48.54) | 352.00 (52.43) |  |
| Female | 6752.00 (51.31) | 6467.00 (51.46) | 285.00 (47.57) |  |
| Age, years (mean (SD)) | 57.665 (14.789) | 57.154 (14.665) | 70.908 (11.458) | <0.0001 |
| Race (%) |  |  |  | <0.0001 |
| Mexican American | 1556.00 (5.41) | 1519.00 (5.50) | 37.00 (2.93) |  |
| Non-Hispanic White | 5998.00 (71.05) | 5593.00 (70.69) | 405.00 (80.39) |  |
| Non-Hispanic Black | 3568.00 (13.67) | 3416.00 (13.70) | 152.00 (12.88) |  |
| Other race | 2108.00 (9.88) | 2065.00 (10.11) | 43.00 (3.80) |  |
| Family poverty-income ratio |  |  |  | <0.0001 |
| <1.3 | 3764.00 (18.98) | 3554.00 (18.68) | 210.00 (26.79) |  |
| 1.3-3.5 | 5934.00 (42.28) | 5608.00 (41.93) | 326.00 (51.25) |  |
| >3.5 | 3532.00 (38.75) | 3431.00 (39.39) | 101.00 (21.96) |  |
| Smoking status (%) |  |  |  | <0.0001 |
| Never | 6649.00 (49.82) | 6365.00 (50.00) | 284.00 (45.11) |  |
| Past | 4156.00 (32.14) | 3904.00 (31.87) | 252.00 (39.02) |  |
| Current | 2425.00 (18.05) | 2324.00 (18.13) | 101.00 (15.87) |  |
| Educational level (%) |  |  |  | <0.0001 |
| High school or less | 6767.00 (42.75) | 6364.00 (42.10) | 403.00 (59.77) |  |
| College or above | 6463.00 (57.25) | 6229.00 (57.90) | 234.00 (40.23) |  |
| Marital status (%) |  |  |  |  |
| Married | 7051.00 (59.08) | 6770.00 (59.47) | 281.00 (48.85) | <0.0001 |
| Widowed | 1820.00 (10.72) | 1616.00 (10.00) | 204.00 (29.27) |  |
| Divorced | 1792.00 (12.59) | 1721.00 (12.70) | 71.00 (9.86) |  |
| Separated | 468.00 (2.51) | 446.00 (2.51) | 22.00 (2.58) |  |
| Never married | 1400.00 (9.40) | 1357.00 (9.50) | 43.00 (6.73) |  |
| Living with partner | 699.00 ( 5.70) | 683.00 (5.82) | 16.00 (2.72) |  |
| BMI, kg/m^2^ (mean (SD)) | 31.169 (7.269) | 31.181 (7.248) | 30.857 (7.784) | 0.4141 |
| Waist circumference, cm (mean (SD)) | 105.712 (16.006) | 105.674 (15.997) | 106.681 (16.232) | 0.244 |
| Total energy intake, kcal/d (mean (SD)) | 1044.656 (478.720) | 1049.720 (479.882) | 913.421 (427.454) | <0.0001 |
| GGT, u/l(mean (SD)) | 34.102 (51.628) | 34.056 (51.925) | 35.279 (43.231) | 0.4994 |
| ALT, u/l(mean (SD)) | 26.323 (20.506) | 26.457 (20.481) | 22.857 (20.860) | 0.0006 |
| ATS, u/l(mean (SD)) | 26.449 (17.380) | 26.433 (17.334) | 26.850 (18.540) | 0.5607 |
| Diabetes (%) |  |  |  | <0.0001 |
| No | 8632.00 (70.93) | 8287.00 (71.51) | 345.00 (56.12) | <0.0001 |
| Yes | 4598.00 (29.07) | 4306.00 (28.49) | 292.00 (43.88) |  |
| CVD (%) |  |  |  | <0.0001 |
| No | 10494.00 (82.59) | 10175.00 (83.77) | 319.00 (51.97) | <0.0001 |
| Yes | 2736.00 (17.41) | 2418.00 (16.23) | 318.00 (48.03) |  |
| CKD (%) |  |  |  | <0.0001 |
| No | 2352.00 (15.27) | 2062.00 (14.12) | 290.00 (45.18) | <0.0001 |
| Yes | 10878.00 (84.73) | 10531.00 (85.88) | 347.00 (54.82) |  |
| Hyperlipidemia (%) |  |  |  | 0.8956 |
| No | 4081.00 (29.10) | 3886.00 (29.09) | 195.00 (29.37) |  |
| Yes | 9149.00 (70.90) | 8707.00 (70.91) | 442.00 (70.63) |  |
| Cancer (%) |  |  |  | <0.0001 |
| No | 11236.00 (83.96) | 10743.00 (84.28) | 493.00 (75.49) | <0.0001 |
| Yes | 1994.00 (16.04) | 1850.00 (15.72) | 144.00 (24.51) |  |
| AHEI (mean (SD)) | 38.956 (11.195) | 38.975 (11.222) | 38.462 (10.480) | 0.376 |
| DASH (Mean (SD) | 22.605 (4.930) | 22.586 (4.936) | 23.081 (4.763) | 0.0549 |
| DII (mean (SD)) | 1.099 (1.675) | 1.090 (1.674) | 1.341 (1.671) | 0.0035 |
| HEI-2020 (mean (SD)) | 51.699 (11.814) | 51.642 (11.809) | 53.164 (11.847) | 0.0323 |
| MED (mean (SD)) | 3.463 (1.338) | 3.464 (1.338) | 3.452 (1.343) | 0.876 |
| MEDI (mean (SD)) | 3.569 (1.016) | 3.572 (1.020) | 3.475 (0.888) | 0.0483 |

^[[1]](#footnote-0)^

1. GGT: Gamma glutamyl transferase; ALT: alanine aminotransferase; AST: aspartate aminotransferase; CVD: cardiovascular disease; CKD: chronic didney disease; AHEI: Alternate Healthy Eating Index; DASH: dietary approaches to stop hypertension index; DII: Dietary Inflammatory Index; HEI-2020: Healthy Eating Index-2020; MED: Mediterranean Diet; MEDI: Mediterranean diet index in serving sizes from the PREDIMED trial. [↑](#footnote-ref-0)
