# Supplementary material for: Associations of dietary indices with risk of all-cause and cardiovascular mortality in hypertensive adults
Source: Ann Med. 2025 Nov 15;57(1):2584427. doi: 10.1080/07853890.2025.2584427 (PMC12621336; doi:10.1080/07853890.2025.2584427)
Supplement: Supplemental Material [file IANN_A_2584427_SM3071.zip › suppl_data/Table S10.docx]

**Table S10.** Hazard Ratios of Mortality According to different dietary indices among hypertensive adults after missing value imputation using K-Nearest Neighbors (KNN).

| Variable | All-cause mortality | | | | Cardiovascular mortality | | | |
| --- | --- | --- | --- | --- | --- | --- | --- | --- |
|  | Model 1 | | Model 2 | | Model 1 | | Model 2 | |
|  | HR (95% CI) | *P* value | HR (95% CI) | *P* value | HR (95% CI) | *P* value | HR (95% CI) | *P* value |
| zAHEI |  |  |  |  |  |  |  |  |
| Continuous | 0.97 (0.92, 1.02) | 0.261 | 0.91 (0.86, 0.97) | 0.002 | 0.91 (0.81, 1.02) | 0.345 | 0.91 (0.81, 1.02) | 0.1 |
| Quartile |  |  |  |  |  |  |  |  |
| Q1 | 1 (Ref) |  | 1 (Ref) |  | 1 (Ref) |  | 1 (Ref) |  |
| Q2 | 1.24 (1.07, 1.43) | 0.005 | 1.03 (0.88, 1.20) | 0.742 | 1.15 (0.88, 1.51) | 0.302 | 0.95 (0.73, 1.25) | 0.734 |
| Q3 | 1.09 (0.93, 1.27) | 0.274 | 0.89 (0.79, 1.01) | 0.077 | 0.97 (0.73, 1.28) | 0.819 | 0.8 (0.61, 1.05) | 0.11 |
| Q4 | 0.98 (0.83, 1.15) | 0.768 | 0.82 (0.70, 0.96) | 0.011 | 0.97 (0.71, 1.32) | 0.827 | 0.84 (0.60, 1.18) | 0.323 |
| *P* value for trend |  | 0.402 |  | 0.003 |  | 0.552 |  | 0.212 |
| zDASH |  |  |  |  |  |  |  |  |
| Continuous | 1.04 (0.98, 1.10) | 0.186 | 0.93 (0.88, 0.98) | 0.011 | 1.06 (0.96, 1.17) | 0.227 | 0.95 (0.85, 1.06) | 0.324 |
| Quartile |  |  |  |  |  |  |  |  |
| Q1 | 1 (Ref) |  | 1 (Ref) |  | 1 (Ref) |  | 1 (Ref) |  |
| Q2 | 1.28 (1.07, 1.52) | 0.006 | 1.01 (0.87, 1.17) | 0.892 | 1.43 (1.05, 1.95) | 0.023 | 1.1 (0.82, 1.49) | 0.522 |
| Q3 | 1.33 (1.11, 1.60) | 0.002 | 0.98 (0.82, 1.17) | 0.809 | 1.26 (0.95, 1.68) | 0.113 | 0.92 (0.70, 1.23) | 0.585 |
| Q4 | 1.22 (1.02, 1.46) | 0.031 | 0.85 (0.72, 1.00) | 0.053 | 1.41 (1.05, 1.89) | 0.021 | 0.97 (0.72, 1.31) | 0.843 |
| *P* value for trend |  | 0.056 |  | 0.033 |  | 0.073 |  | 0.537 |
| zDII |  |  |  |  |  |  |  |  |
| Continuous | 1.21 (1.14, 1.28) | <0.001 | 1.12 (1.05, 1.21) | 0.001 | 1.19 (1.07, 1.33) | 0.001 | 1.09 (0.95, 1.24) | 0.216 |
| Quartile |  |  |  |  |  |  |  |  |
| Q1 | 1 (Ref) |  | 1 (Ref) |  | 1 (Ref) |  | 1 (Ref) |  |
| Q2 | 1.16 (0.98, 1.38) | 0.089 | 1.09 (0.92, 1.30) | 0.326 | 1.22 (0.91, 1.65) | 0.185 | 1.12 (0.84, 1.48) | 0.445 |
| Q3 | 1.28 (1.09, 1.51) | 0.002 | 1.1 (0.92, 1.31) | 0.283 | 1.32 (0.98, 1.76) | 0.064 | 1.1 (0.78, 1.57) | 0.588 |
| Q4 | 1.63 (1.40, 1.91) | <0.001 | 1.31 (1.07, 1.60) | 0.009 | 1.64 (1.24, 2.16) | <0.001 | 1.26 (0.90, 1.78) | 0.184 |
| *P* value for trend |  | <0.001 |  | 0.01 |  | <0.001 |  | 0.239 |
| zHEI-2020 |  |  |  |  |  |  |  |  |
| Continuous | 1.05 (0.99, 1.11) | 0.085 | 0.92 (0.87, 0.98) | 0.006 | 1.11 (0.99, 1.25) | 0.069 | 0.98 (0.86, 1.11) | 0.717 |
| Quartile |  |  |  |  |  |  |  |  |
| Q1 | 1 (Ref) |  | 1 (Ref) |  | 1 (Ref) |  | 1 (Ref) |  |
| Q2 | 1.17 (1.03, 1.33) | 0.013 | 0.99 (0.86, 1.15) | 0.909 | 1.17 (0.89, 1.54) | 0.25 | 0.97 (0.72, 1.30) | 0.831 |
| Q3 | 1.26 (1.09, 1.45) | 0.001 | 0.96 (0.83, 1.12) | 0.62 | 1.3 (0.99, 1.71) | 0.062 | 0.95 (0.72, 1.26) | 0.744 |
| Q4 | 1.17 (0.99, 1.38) | 0.058 | 0.81 (0.69, 0.96) | 0.016 | 1.29 (0.95, 1.76) | 0.108 | 0.9 (0.66, 1.23) | 0.505 |
| *P* value for trend |  | 0.036 |  | 0.008 |  | 0.096 |  | 0.523 |
| zMED |  |  |  |  |  |  |  |  |
| Continuous | 0.94 (0.89, 1.00) | 0.049 | 0.9 (0.86, 0.96) | <0.001 | 0.96 (0.87, 1.07) | 0.475 | 0.92 (0.82, 1.04) | 0.179 |
| Quartile |  |  |  |  |  |  |  |  |
| Q1 | 1 (Ref) |  | 1 (Ref) |  | 1 (Ref) |  | 1 (Ref) |  |
| Q2 | 0.92 (0.78, 1.08) | 0.287 | 0.87 (0.75, 1.01) | 0.074 | 0.91 (0.68, 1.23) | 0.553 | 0.85 (0.63, 1.15) | 0.306 |
| Q3 | 0.92 (0.78, 1.08) | 0.297 | 0.84 (0.73, 0.97) | 0.019 | 0.91 (0.66, 1.25) | 0.562 | 0.8 (0.58, 1.11) | 0.18 |
| Q4 | 0.84 (0.71, 1.00) | 0.052 | 0.75 (0.63, 0.88) | <0.001 | 0.85 (0.62, 1.17) | 0.312 | 0.74 (0.53, 1.05) | 0.088 |
| *P* value for trend |  | 0.07 |  | <0.001 |  | 0.33 |  | 0.085 |
| zMEDI |  |  |  |  |  |  |  |  |
| Continuous | 0.94 (0.90, 0.99) | 0.01 | 0.94 (0.89, 0.99) | 0.012 | 0.91 (0.83, 1.00) | 0.053 | 0.93 (0.84, 1.04) | 0.192 |
| Quartile |  |  |  |  |  |  |  |  |
| Q1 | 1 (Ref) |  | 1 (Ref) |  | 1 (Ref) |  | 1 (Ref) |  |
| Q2 | 1.47 (1.23, 1.75) | <0.001 | 1.11 (0.94, 1.31) | 0.219 | 1.46 (1.06, 2.01) | 0.02 | 1.04 (0.77, 1.42) | 0.78 |
| Q3 | 1.4 (1.18, 1.65) | <0.001 | 1.02 (0.88, 1.19) | 0.756 | 1.27 (0.90, 1.81) | 0.179 | 0.91 (0.65, 1.28) | 0.587 |
| Q4 | 1.07 (0.92, 1.24) | 0.401 | 0.96 (0.84, 1.11) | 0.62 | 1.01 (0.75, 1.37) | 0.942 | 0.95 (0.70, 1.29) | 0.758 |
| *P* value for trend |  | 0.631 |  | 0.268 |  | 0.47 |  | 0.575 |

^[[1]](#footnote-0)^

1. HR= hazard ratio; CI= confidence interval. Model 1 was unadjusted; Model 2 was adjusted for sex, age, race, educational level, family poverty-income ratio, marital status, smoking status, BMI, waist circumference, GGT, AST, ALT, total energy intake, diabetes, CVD, CKD, hyperlipidemia, and cancer. [↑](#footnote-ref-0)
