# Supplementary material for: Associations of dietary indices with risk of all-cause and cardiovascular mortality in hypertensive adults
Source: Ann Med. 2025 Nov 15;57(1):2584427. doi: 10.1080/07853890.2025.2584427 (PMC12621336; doi:10.1080/07853890.2025.2584427)
Supplement: Supplemental Material [file IANN_A_2584427_SM3071.zip › suppl_data/Table S11.docx]

**Table S11.** Assessment of the Combined effects of Dietary Patterns on all-cause and cardiovascular deaths Using WQS Regression

|  | All-cause mortality | | Cardiovascular mortality | |
| --- | --- | --- | --- | --- |
|  | HR (95%CI) | *P* | HR (95%CI) | *P* |
| AHEI | 0.986 (0.909, 1.070) | 0.742 | 0.991(0.856, 1.147) | 0.9044 |
| DASH | 0.940 (0.868, 1.017) | 0.123 | 0.831 (0.711, 0.971) | 0.0195 |
| DII | 1.237 (1.134, 1.351) | <0.001 | 1.078(0.902,1.288) | 0.4061 |
| HEI-2020 | 0.929 (0.893, 0.966) | <0.001 | 0.883 (0.797, 0.979) | 0.0171 |
| MED | 0.964 (0.683, 1.360) | 0.834 | 0.840 (0.504, 1.396) | 0.4998 |
| MEDI | 0.513 (0.327, 0.804) | 0.003 | 0.622 (0.316, 1.225) | 0.1695 |

^[[1]](#footnote-0)^

1. HR= hazard ratio; CI= confidence interval. Model was adjusted for sex, age, race, educational level, family poverty-income ratio, marital status, smoking status, BMI, waist circumference, GGT, AST, ALT, total energy intake, diabetes, CVD, CKD, hyperlipidemia, and cancer. [↑](#footnote-ref-0)
