# Supplementary material for: Associations of dietary indices with risk of all-cause and cardiovascular mortality in hypertensive adults
Source: Ann Med. 2025 Nov 15;57(1):2584427. doi: 10.1080/07853890.2025.2584427 (PMC12621336; doi:10.1080/07853890.2025.2584427)
Supplement: Supplemental Material [file IANN_A_2584427_SM3071.zip › suppl_data/Table S2.docx]

**Table S2.** Hazard Ratios of Mortality According to different dietary indices among hypertensive adults after propensity score matching

| Variable | All-cause mortality | | | | Cardiovascular mortality | | | |
| --- | --- | --- | --- | --- | --- | --- | --- | --- |
|  | Model 1 | | Model 2 | | Model 1 | | Model 2 | |
|  | HR (95% CI) | *P* value | HR (95% CI) | *P* value | HR (95% CI) | *P* value | HR (95% CI) | *P* value |
| zAHEI |  |  |  |  |  |  |  |  |
| Continuous | 0.94 (0.88, 1.00) | 0.057 | 0.9 (0.85, 0.95) | <0.001 | 0.9 (0.81, 1.01) | 0.078 | 0.88 (0.78, 0.99) | 0.032 |
| Quartile |  |  |  |  |  |  |  |  |
| Q1 | 1 (Ref) |  | 1 (Ref) |  | 1 (Ref) |  | 1 (Ref) |  |
| Q2 | 1.21 (1.06, 1.40) | 0.006 | 1.08 ( 0.93, 1.24) | 0.971 | 1.13 (0.85, 1.51) | 0.39 | 1.01 (0.75, 1.35) | 0.969 |
| Q3 | 1 (0.85, 1.17) | 0.971 | 0.87 ( 0.77, 0.99) | 0.038 | 0.87 (0.64, 1.19) | 0.385 | 0.76 (0.56, 1.02) | 0.069 |
| Q4 | 0.94 ( 0.80, 1.11) | 0.491 | 0.84 (0.72, 0.97) | 0.017 | 0.87 (0.62, 1.23) | 0.446 | 0.82 (0.57, 1.16) | 0.257 |
| *P* value for trend |  | 0.152 |  | 0.003 |  | 0.218 |  | 0.11 |
| zDASH |  |  |  |  |  |  |  |  |
| Continuous | 1.01 (0.95, 1.08) | 0.732 | 0.94 (0.88, 0.99) | 0.029 | 1.06 (0.94, 1.18) | 0.359 | 0.96 (0.85, 1.08) | 0.509 |
| Quartile |  |  |  |  |  |  |  |  |
| Q1 | 1 (Ref) |  | 1 (Ref) |  | 1 (Ref) |  | 1 (Ref) |  |
| Q2 | 1.39 (1.14, 1.70) | 0.001 | 1.12 (0.95, 1.32) | 0.168 | 1.64 (1.17, 2.31) | 0.004 | 1.27 (0.89, 1.79) | 0.183 |
| Q3 | 1.36 (1.13, 1.65) | 0.001 | 1.06 (0.88, 1.29) | 0.518 | 1.37 (0.99, 1.89) | 0.059 | 1.06 (0.78, 1.45) | 0.7 |
| Q4 | 1.15 (0.94, 1.42) | 0.18 | 0.9 (0.76, 1.06) | 0.201 | 1.5 (1.07, 2.11) | 0.019 | 1.11 (0.79, 1.55) | 0.543 |
| *P* value for trend |  | 0.556 |  | 0.037 |  | 0.138 |  | 0.96 |
| zDII |  |  |  |  |  |  |  |  |
| Continuous | 1.14 (1.05, 1.24) | 0.001 | 1.14 (1.05, 1.25) | 0.002 | 1.18 (1.01, 1.37) | 0.037 | 1.15 (0.98, 1.35) | 0.081 |
| Quartile |  |  |  |  |  |  |  |  |
| Q1 | 1 (Ref) |  | 1 (Ref) |  | 1 (Ref) |  | 1 (Ref) |  |
| Q2 | 1.06 (0.88, 1.27) | 0.567 | 1.08 (0.89, 1.32) | 0.429 | 1.23 (0.85, 1.77) | 0.265 | 1.24 (0.85, 1.82) | 0.259 |
| Q3 | 1.03 (0.83, 1.28) | 0.792 | 1.09 (0.88, 1.35) | 0.441 | 1.09 ( 0.74, 1.62) | 0.654 | 1.13 (0.76, 1.70) | 0.543 |
| Q4 | 1.36 (1.15, 1.60) | <0.001 | 1.33 (1.09, 1.63) | 0.005 | 1.56 (1.11, 2.20) | 0.011 | 1.46 (1.00, 2.13) | 0.053 |
| *P* value for trend |  | 0.001 |  | 0.006 |  | 0.03 |  | 0.089 |
| zHEI-2020 |  |  |  |  |  |  |  |  |
| Continuous | 1.03 (0.96, 1.09) | 0.453 | 0.94 (0.88, 1.00) | 0.051 | 1.05 (0.93, 1.19) | 0.416 | 0.96 (0.85, 1.10) | 0.574 |
| Quartile |  |  |  |  |  |  |  |  |
| Q1 | 1 (Ref) |  | 1 (Ref) |  | 1 (Ref) |  | 1 (Ref) |  |
| Q2 | 1.17 (0.99, 1.38) | 0.064 | 1.04 (0.87, 1.25) | 0.646 | 1.23 (0.90, 1.69) | 0.198 | 1.06 (0.76, 1.48) | 0.75 |
| Q3 | 1.1 (0.94, 1.29) | 0.219 | 0.98 (0.83, 1.15) | 0.791 | 1.19 (0.90, 1.56) | 0.227 | 1.02 (0.76, 1.37) | 0.904 |
| Q4 | 1.09 (0.91, 1.31) | 0.331 | 0.87 (0.72, 1.05) | 0.136 | 1.16 (0.85, 1.58) | 0.356 | 0.91 (0.65, 1.27) | 0.574 |
| *P* value for trend |  | 0.523 |  | 0.049 |  | 0.474 |  | 0.494 |
| zMED |  |  |  |  |  |  |  |  |
| Continuous | 0.98 (0.92, 1.05) | 0.605 | 0.92 (0.87, 0.97) | 0.005 | 0.97 (0.86, 1.09) | 0.633 | 0.9 (0.80, 1.02) | 0.092 |
| Quartile |  |  |  |  |  |  |  |  |
| Q1 | 1 (Ref) |  | 1 (Ref) |  | 1 (Ref) |  | 1 (Ref) |  |
| Q2 | 0.85 (0.70, 1.03) | 0.092 | 0.81 (0.68, 0.96) | 0.018 | 0.82 (0.56, 1.18) | 0.279 | 0.78 (0.54, 1.12) | 0.175 |
| Q3 | 0.92 (0.74, 1.14) | 0.44 | 0.89 (0.76, 1.03) | 0.124 | 0.86 (0.58, 1.27) | 0.44 | 0.79 (0.55, 1.14) | 0.2 |
| Q4 | 0.92 (0.77, 1.10) | 0.37 | 0.76 (0.66, 0.89) | <0.001 | 0.88 (0.61, 1.27) | 0.504 | 0.71 (0.50, 1.00) | 0.053 |
| *P* value for trend |  | 0.822 |  | 0.006 |  | 0.725 |  | 0.061 |
| zMEDI |  |  |  |  |  |  |  |  |
| Continuous | 0.97 (0.92, 1.02) | 0.207 | 0.95 (0.90, 1.00) | 0.067 | 0.94 (0.84, 1.04) | 0.22 | 0.92 (0.81, 1.03) | 0.156 |
| Quartile |  |  |  |  |  |  |  |  |
| Q1 | 1 (Ref) |  | 1 (Ref) |  | 1 (Ref) |  | 1 (Ref) |  |
| Q2 | 1.43 (1.15, 1.77) | 0.001 | 1.2 (0.97, 1.48) | 0.096 | 1.48 (0.92, 2.36) | 0.103 | 1.13 (0.70, 1.83) | 0.607 |
| Q3 | 1.44 (1.11, 1.86) | 0.006 | 1.06 (0.83, 1.35) | 0.648 | 1.5 (0.87, 2.59) | 0.144 | 1 (0.59, 1.70) | 0.994 |
| Q4 | 1.18 (0.94, 1.49) | 0.142 | 1.07 (0.86, 1.32) | 0.538 | 1.16 (0.70, 1.90) | 0.57 | 0.99 (0.60, 1.61) | 0.961 |
| *P* value for trend |  | 0.526 |  | 0.385 |  | 0.536 |  | 0.458 |

^[[1]](#footnote-0)^

1. HR= hazard ratio; CI= confidence interval. Model 1 was unadjusted; Model 2 was adjusted for sex, age, race, educational level, family poverty-income ratio, marital status, smoking status, BMI, waist circumference, GGT, AST, ALT, total energy intake, diabetes, CVD, CKD, hyperlipidemia, and cancer. [↑](#footnote-ref-0)
