# Supplementary material for: Associations of dietary indices with risk of all-cause and cardiovascular mortality in hypertensive adults
Source: Ann Med. 2025 Nov 15;57(1):2584427. doi: 10.1080/07853890.2025.2584427 (PMC12621336; doi:10.1080/07853890.2025.2584427)
Supplement: Supplemental Material [file IANN_A_2584427_SM3071.zip › suppl_data/Table S3.docx]

**Table S3.** Hazard Ratios of Mortality According to different dietary indices among hypertensive adults after excluding participants with CVD or cancer at baseline.

| Variable | All-cause mortality | | | | Cardiovascular mortality | | | |
| --- | --- | --- | --- | --- | --- | --- | --- | --- |
|  | Model 1 | | Model 2 | | Model 1 | | Model 2 | |
|  | HR (95% CI) | *P* value | HR (95% CI) | *P* value | HR (95% CI) | *P* value | HR (95% CI) | *P* value |
| zAHEI |  |  |  |  |  |  |  |  |
| Continuous | 0.94 (0.88, 1.02) | 0.13 | 0.9 (0.83, 0.98) | 0.014 | 0.93 (0.80, 1.08) | 0.335 | 0.92 (0.75, 1.13) | 0.422 |
| Quartile |  |  |  |  |  |  |  |  |
| Q1 | 1 (Ref) |  | 1 (Ref) |  | 1 (Ref) |  | 1 (Ref) |  |
| Q2 | 1.31 (1.02, 1.67) | 0.032 | 1.09 (0.85, 1.41) | 0.497 | 1.17 (0.71, 1.92) | 0.537 | 0.98 (0.59, 1.62) | 0.931 |
| Q3 | 1.13 (0.91, 1.42) | 0.266 | 0.95 (0.76, 1.17) | 0.611 | 0.88 (0.58, 1.33) | 0.543 | 0.74 (0.46, 1.18) | 0.203 |
| Q4 | 0.89 (0.69, 1.13) | 0.341 | 0.77 (0.60, 0.98) | 0.033 | 0.86 (0.51, 1.46) | 0.589 | 0.84 (0.47, 1.52) | 0.57 |
| *P* value for trend |  | 0.204 |  | 0.018 |  | 0.38 |  | 0.415 |
| zDASH |  |  |  |  |  |  |  |  |
| Continuous | 1.02 (0.95, 1.09) | 0.628 | 0.94 (0.86, 1.01) | 0.098 | 1 (0.86, 1.16) | 0.955 | 0.94 (0.77, 1.16) | 0.579 |
| Quartile |  |  |  |  |  |  |  |  |
| Q1 | 1 (Ref) |  | 1 (Ref) |  | 1 (Ref) |  | 1 (Ref) |  |
| Q2 | 1.25 (0.95, 1.64) | 0.112 | 1.12 (0.81, 1.29) | 0.881 | 1.77 (1.10, 2.83) | 0.018 | 1.45 (0.91, 2.30) | 0.114 |
| Q3 | 1.32 (1.03, 1.69) | 0.026 | 1.07 (0.84, 1.38) | 0.582 | 1.23 (0.80, 1.88) | 0.35 | 1.05 (0.66, 1.69) | 0.823 |
| Q4 | 1.15 (0.91, 1.46) | 0.245 | 0.86 (0.68, 1.09) | 0.224 | 1.28 (0.80, 2.06) | 0.303 | 1.01 (0.58, 1.76) | 0.968 |
| *P* value for trend |  | 0.228 |  | 0.229 |  | 0.764 |  | 0.589 |
| zDII |  |  |  |  |  |  |  |  |
| Continuous | 1.27 (1.16, 1.39) | <0.001 | 1.17 (1.05, 1.30) | 0.004 | 1.25 (1.04, 1.51) | 0.019 | 1.11 (0.88, 1.39) | 0.365 |
| Quartile |  |  |  |  |  |  |  |  |
| Q1 | 1 (Ref) |  | 1 (Ref) |  | 1 (Ref) |  | 1 (Ref) |  |
| Q2 | 1.33 (1.02, 1.75) | 0.037 | 1.28 (0.99, 1.66) | 0.064 | 1.43 (0.88, 2.31) | 0.148 | 1.29 (0.80, 2.08) | 0.298 |
| Q3 | 1.48 (1.18, 1.86) | <0.001 | 1.22 (0.96, 1.54) | 0.11 | 1.3 (0.82, 2.06) | 0.262 | 1.01 (0.60, 1.69) | 0.982 |
| Q4 | 1.87 (1.44, 2.41) | <0.001 | 1.49 (1.09, 2.05) | 0.013 | 2.17 (1.37, 3.43) | <0.001 | 1.67 (0.93, 3.01) | 0.087 |
| *P* value for trend |  | <0.001 |  | 0.019 |  | 0.003 |  | 0.18 |
| zHEI-2020 |  |  |  |  |  |  |  |  |
| Continuous | 1.02 (0.94, 1.11) | 0.668 | 0.91 (0.83, 0.98) | 0.017 | 1.05 (0.86, 1.27) | 0.639 | 0.98 (0.79, 1.22) | 0.856 |
| Quartile |  |  |  |  |  |  |  |  |
| Q1 | 1 (Ref) |  | 1 (Ref) |  | 1 (Ref) |  | 1 (Ref) |  |
| Q2 | 1.14 (0.94, 1.38) | 0.181 | 1 (0.82, 1.21) | 0.985 | 1 (0.65, 1.53) | 0.997 | 0.85 (0.55, 1.33) | 0.484 |
| Q3 | 1.14 (0.91, 1.42) | 0.264 | 0.93 (0.73, 1.18) | 0.541 | 0.89 (0.53, 1.49) | 0.665 | 0.75 (0.43, 1.30) | 0.308 |
| Q4 | 1.08 (0.86, 1.37) | 0.504 | 0.79 (0.63, 0.98) | 0.03 | 1.07 (0.65, 1.76) | 0.789 | 0.85 (0.53, 1.38) | 0.52 |
| *P* value for trend |  | 0.533 |  | 0.021 |  | 0.913 |  | 0.492 |
| zMED |  |  |  |  |  |  |  |  |
| Continuous | 0.92 (0.85, 0.99) | 0.029 | 0.91 (0.84, 0.99) | 0.03 | 0.97 (0.86, 1.09) | 0.633 | 0.9 (0.80, 1.02) | 0.092 |
| Quartile |  |  |  |  |  |  |  |  |
| Q1 | 1 (Ref) |  | 1 (Ref) |  | 1 (Ref) |  | 1 (Ref) |  |
| Q2 | 0.9 (0.71, 1.13) | 0.364 | 0.91 (0.72, 1.15) | 0.441 | 0.71 (0.44, 1.15) | 0.168 | 0.73 (0.44, 1.22) | 0.232 |
| Q3 | 0.88 (0.69, 1.13) | 0.32 | 0.87 (0.68, 1.11) | 0.268 | 0.6 (0.35, 1.02) | 0.058 | 0.6 (0.34, 1.09) | 0.093 |
| Q4 | 0.78 (0.61, 0.99) | 0.041 | 0.79 (0.62, 1.01) | 0.063 | 0.72 (0.43, 1.20) | 0.203 | 0.81 (0.44, 1.47) | 0.484 |
| *P* value for trend |  | 0.056 |  | 0.066 |  | 0.205 |  | 0.438 |
| zMEDI |  |  |  |  |  |  |  |  |
| Continuous | 0.99 (0.92, 1.06) | 0.768 | 0.97 (0.90, 1.05) | 0.488 | 0.95 (0.81, 1.12) | 0.519 | 0.98 (0.81, 1.17) | 0.789 |
| Quartile |  |  |  |  |  |  |  |  |
| Q1 | 1 (Ref) |  | 1 (Ref) |  | 1 (Ref) |  | 1 (Ref) |  |
| Q2 | 1.62 (1.26, 2.08) | <0.001 | 1.29 (1.00, 1.65) | 0.046 | 1.4 (0.84, 2.36) | 0.199 | 1.07 (0.61, 1.88) | 0.804 |
| Q3 | 1.63 (1.27, 2.09) | <0.001 | 1.21 (0.94, 1.55) | 0.142 | 1.32 (0.77, 2.26) | 0.308 | 0.96 (0.57, 1.62) | 0.876 |
| Q4 | 1.25 (0.99, 1.59) | 0.066 | 1.11 (0.88, 1.38) | 0.382 | 1.09 (0.68, 1.76) | 0.72 | 1.08 (0.66, 1.75) | 0.767 |
| *P* value for trend |  | 0.284 |  | 0.796 |  | 0.975 |  | 0.846 |

^[[1]](#footnote-0)^

1. HR= hazard ratio; CI= confidence interval. Model 1 was unadjusted; Model 2 was adjusted for sex, age, race, educational level, family poverty-income ratio, marital status, smoking status, BMI, waist circumference, GGT, AST, ALT, total energy intake, diabetes, CKD, and hyperlipidemia. [↑](#footnote-ref-0)
