# Supplementary material for: Associations of dietary indices with risk of all-cause and cardiovascular mortality in hypertensive adults
Source: Ann Med. 2025 Nov 15;57(1):2584427. doi: 10.1080/07853890.2025.2584427 (PMC12621336; doi:10.1080/07853890.2025.2584427)
Supplement: Supplemental Material [file IANN_A_2584427_SM3071.zip › suppl_data/Table S4.docx]

**Table S4A.** Hazard Ratios of Mortality According to different dietary indices among hypertensive adults after excluding participants who died within 1 year of follow-up.

| Variable | All-cause mortality | | | | Cardiovascular mortality | | | |
| --- | --- | --- | --- | --- | --- | --- | --- | --- |
|  | Model 1 | | Model 2 | | Model 1 | | Model 2 | |
|  | HR (95% CI) | *P* value | HR (95% CI) | *P* value | HR (95% CI) | *P* value | HR (95% CI) | *P* value |
| zAHEI |  |  |  |  |  |  |  |  |
| Continuous | 0.99 (0.93, 1.04) | 0.647 | 0.92 (0.87, 0.97) | 0.004 | 0.96 (0.87, 1.06) | 0.407 | 0.89 (0.79, 1.01) | 0.067 |
| Quartile |  |  |  |  |  |  |  |  |
| Q1 | 1 (Ref) |  | 1 (Ref) |  | 1 (Ref) |  | 1 (Ref) |  |
| Q2 | 1.22 (1.06, 1.41) | 0.006 | 1.01 (0.86, 1.18) | 0.913 | 1.15 (0.88, 1.48) | 0.302 | 0.95 (0.73, 1.23) | 0.689 |
| Q3 | 1.13 (0.97, 1.32) | 0.118 | 0.92 (0.81, 1.05) | 0.201 | 0.97 (0.74, 1.28) | 0.836 | 0.79 (0.60, 1.04) | 0.091 |
| Q4 | 1.02 (0.87, 1.21) | 0.788 | 0.83 (0.71, 0.97) | 0.022 | 1 (0.73, 1.37) | 0.996 | 0.84 (0.59, 1.19) | 0.326 |
| *P* value for trend |  | 0.901 |  | 0.011 |  | 0.729 |  | 0.219 |
| zDASH |  |  |  |  |  |  |  |  |
| Continuous | 1.05 (0.99, 1.11) | 0.082 | 0.92 (0.87, 0.97) | 0.004 | 1.07 (0.97, 1.17) | 0.175 | 0.93 (0.84, 1.03) | 0.163 |
| Quartile |  |  |  |  |  |  |  |  |
| Q1 | 1 (Ref) |  | 1 (Ref) |  | 1 (Ref) |  | 1 (Ref) |  |
| Q2 | 1.34 (1.13, 1.60) | <0.001 | 1.04 (0.90, 1.20) | 0.626 | 1.4 (1.02, 1.93) | 0.039 | 1.03 (0.76, 1.39) | 0.861 |
| Q3 | 1.44 (1.19, 1.73) | <0.001 | 1.03 (0.86, 1.24) | 0.745 | 1.3 (0.96, 1.75) | 0.093 | 0.92 (0.68, 1.24) | 0.574 |
| Q4 | 1.28 (1.07, 1.53) | 0.008 | 0.85 (0.72, 1.00) | 0.052 | 1.45 (1.08, 1.95) | 0.013 | 0.94 (0.69, 1.29) | 0.697 |
| *P* value for trend |  | 0.015 |  | 0.031 |  | 0.03 |  | 0.545 |
| zDII |  |  |  |  |  |  |  |  |
| Continuous | 1.2 (1.13, 1.27) | <0.001 | 1.13 (1.05, 1.22) | <0.001 | \| 1.19 (1.07, 1.33) \| \| --- \| | 0.001 | 1.11 (0.96, 1.28) | 0.156 |
| Quartile |  |  |  |  |  |  |  |  |
| Q1 | 1 (Ref) |  | 1 (Ref) |  | 1 (Ref) |  | 1 (Ref) |  |
| Q2 | 1.15 (0.97, 1.37) | 0.116 | 1.11 (0.93, 1.32) | 0.265 | 1.21 (0.90, 1.63) | 0.215 | 1.13 (0.85, 1.50) | 0.4 |
| Q3 | 1.23 (1.04, 1.46) | 0.015 | 1.1 (0.91, 1.32) | 0.338 | 1.3 (0.96, 1.75) | 0.095 | 1.12 (0.77, 1.62) | 0.544 |
| Q4 | 1.6 (1.36, 1.88) | <0.001 | 1.33 (1.08, 1.64) | 0.007 | 1.59 (1.19, 2.11) | 0.002 | 1.27 (0.88, 1.82) | 0.2 |
| *P* value for trend |  | <0.001 |  | 0.009 |  | 0.001 |  | 0.25 |
| zHEI-2020 |  |  |  |  |  |  |  |  |
| Continuous | 1.06 (1.01, 1.12) | 0.025 | 0.92 (0.87, 0.98) | 0.005 | 1.11 (1.00, 1.24) | 0.055 | 0.96 (0.85, 1.09) | 0.55 |
| Quartile |  |  |  |  |  |  |  |  |
| Q1 | 1 (Ref) |  | 1 (Ref) |  | 1 (Ref) |  | 1 (Ref) |  |
| Q2 | 1.24 (1.09, 1.40) | <0.001 | 1.04 (0.90, 1.20) | 0.611 | 1.21 (0.91, 1.60) | 0.192 | 0.98 (0.72, 1.33) | 0.902 |
| Q3 | 1.29 (1.13, 1.49) | <0.001 | 0.98 (0.84, 1.14) | 0.814 | 1.32 (1.02, 1.72) | 0.037 | 0.97 (0.74, 1.26) | 0.797 |
| Q4 | 1.22 (1.04, 1.43) | 0.017 | 0.82 (0.70, 0.97) | 0.023 | 1.32 (0.97, 1.79) | 0.078 | 0.89 (0.65, 1.20) | 0.435 |
| *P* value for trend |  | 0.016 |  | 0.009 |  | 0.076 |  | 0.454 |
| zMED |  |  |  |  |  |  |  |  |
| Continuous | 0.96 (0.91, 1.02) | 0.189 | 0.91 (0.86, 0.96) | 0.001 | 0.98 (0.88, 1.09) | 0.714 | 0.93 (0.83, 1.04) | 0.2 |
| Quartile |  |  |  |  |  |  |  |  |
| Q1 | 1 (Ref) |  | 1 (Ref) |  | 1 (Ref) |  | 1 (Ref) |  |
| Q2 | 1 (0.83, 1.20) | 0.988 | 0.94 (0.79, 1.12) | 0.503 | 0.99 (0.72, 1.36) | 0.944 | 0.91 (0.66, 1.25) | 0.557 |
| Q3 | 1 (0.84, 1.19) | 0.975 | 0.91 (0.78, 1.05) | 0.185 | 1.01 (0.73, 1.40) | 0.962 | 0.87 (0.63, 1.19) | 0.378 |
| Q4 | 0.91 (0.76, 1.08) | 0.281 | 0.78 (0.66, 0.92) | 0.004 | 0.92 (0.67, 1.26) | 0.591 | 0.78 (0.55, 1.09) | 0.148 |
| *P* value for trend |  | 0.252 |  | 0.003 |  | 0.593 |  | 0.124 |
| zMEDI |  |  |  |  |  |  |  |  |
| Continuous | 0.95 (0.92, 1.00) | 0.031 | 0.94 (0.90, 0.99) | 0.025 | 0.94 (0.86, 1.02) | 0.119 | 0.95 (0.86, 1.05) | 0.289 |
| Quartile |  |  |  |  |  |  |  |  |
| Q1 | 1 (Ref) |  | 1 (Ref) |  | 1 (Ref) |  | 1 (Ref) |  |
| Q2 | 1.47 (1.22, 1.77) | <0.001 | 1.1 (0.92, 1.31) | 0.303 | 1.51 (1.10, 2.07) | 0.012 | 1.04 (0.76, 1.43) | 0.802 |
| Q3 | 1.44 (1.21, 1.71) | <0.001 | 1.04 (0.89, 1.21) | 0.655 | 1.37 (0.98, 1.90) | 0.066 | 0.95 (0.69, 1.32) | 0.765 |
| Q4 | 1.1 (0.95, 1.27) | 0.217 | 0.97 (0.84, 1.12) | 0.702 | 1.1 (0.83, 1.46) | 0.517 | 1.01 (0.76, 1.34) | 0.961 |
| *P* value for trend |  | 0.964 |  | 0.362 |  | 0.897 |  | 0.914 |

^[[1]](#footnote-0)^

**Table S4B.** Hazard Ratios of Mortality According to different dietary indices among hypertensive adults after excluding participants who died within 2 years of follow-up.

| Variable | All-cause mortality | | | | Cardiovascular mortality | | | |
| --- | --- | --- | --- | --- | --- | --- | --- | --- |
|  | Model 1 | | Model 2 | | Model 1 | | Model 2 | |
|  | HR (95% CI) | *P* value | HR (95% CI) | *P* value | HR (95% CI) | *P* value | HR (95% CI) | *P* value |
| zAHEI |  |  |  |  |  |  |  |  |
| Continuous | 1 (0.95, 1.06) | 0.878 | 0.94 (0.88, 1.00) | 0.044 | 0.98 (0.89, 1.09) | 0.762 | 0.91 (0.80, 1.04) | 0.15 |
| Quartile |  |  |  |  |  |  |  |  |
| Q1 | 1 (Ref) |  | 1 (Ref) |  | 1 (Ref) |  | 1 (Ref) |  |
| Q2 | 1.25 (1.06, 1.47) | 0.007 | 1.03 (0.86, 1.23) | 0.768 | 1.19 (0.89, 1.59) | 0.235 | 0.97 (0.73, 1.30) | 0.86 |
| Q3 | 1.14 (0.96, 1.34) | 0.138 | 0.92 (0.79, 1.06) | 0.244 | 0.99 (0.73, 1.35) | 0.949 | 0.79 (0.58, 1.07) | 0.129 |
| Q4 | 1.07 (0.90, 1.28) | 0.42 | 0.87 (0.72, 1.04) | 0.133 | 1.11 (0.79, 1.57) | 0.543 | 0.91 (0.62, 1.33) | 0.63 |
| *P* value for trend |  | 0.75 |  | 0.063 |  | 0.808 |  | 0.448 |
| zDASH |  |  |  |  |  |  |  |  |
| Continuous | 1.06 (1.00, 1.13) | 0.037 | 0.93 (0.87, 0.99) | 0.033 | 1.08 (0.98, 1.20) | 0.113 | 0.93 (0.83, 1.05) | 0.238 |
| Quartile |  |  |  |  |  |  |  |  |
| Q1 | 1 (Ref) |  | 1 (Ref) |  | 1 (Ref) |  | 1 (Ref) |  |
| Q2 | 1.25 (1.06, 1.47) | 0.007 | 1.03 (0.86, 1.23) | 0.768 | 1.49 (1.03, 2.13) | 0.032 | 1.06 (0.76, 1.50) | 0.726 |
| Q3 | 1.14 (0.96, 1.34) | 0.138 | 0.92 (0.79, 1.06) | 0.244 | 1.31 (0.95, 1.80) | 0.102 | 0.9 (0.66, 1.21) | 0.476 |
| Q4 | 1.07 (0.90, 1.28) | 0.42 | 0.87 (0.72, 1.04) | 0.133 | 1.54 (1.10, 2.16) | 0.011 | 0.96 (0.67, 1.36) | 0.811 |
| *P* value for trend |  | 0.75 |  | 0.063 |  | 0.025 |  | 0.578 |
| zDII |  |  |  |  |  |  |  |  |
| Continuous | 1.17 (1.11, 1.24) | <0.001 | 1.11 (1.03, 1.20) | 0.006 | 1.15 (1.03, 1.29) | 0.014 | 1.09 (0.94, 1.26) | 0.271 |
| Quartile |  |  |  |  |  |  |  |  |
| Q1 | 1 (Ref) |  | 1 (Ref) |  | 1 (Ref) |  | 1 (Ref) |  |
| Q2 | 1.15 (0.95, 1.38) | 0.144 | 1.1 (0.92, 1.33) | 0.294 | 1.17 (0.86, 1.61) | 0.323 | 1.11 (0.82, 1.51) | 0.5 |
| Q3 | 1.2 (1.00, 1.44) | 0.053 | 1.06 (0.87, 1.30) | 0.558 | 1.25 (0.91, 1.70) | 0.168 | 1.1 (0.75, 1.61) | 0.634 |
| Q4 | 1.52 (1.30, 1.79) | <0.001 | 1.27 (1.02, 1.58) | 0.029 | 1.45 (1.07, 1.96) | 0.017 | 1.2 (0.81, 1.76) | 0.365 |
| *P* value for trend |  | <0.001 |  | 0.051 |  | 0.014 |  | 0.423 |
| zHEI-2020 |  |  |  |  |  |  |  |  |
| Continuous | 1.08 (1.02, 1.15) | 0.005 | 0.94 (0.88, 1.00) | 0.036 | 1.13 (1.01, 1.27) | \| 0.033 \| \| --- \| | 0.97 (0.85, 1.10) | 0.646 |
| Quartile |  |  |  |  |  |  |  |  |
| Q1 | 1 (Ref) |  | 1 (Ref) |  | 1 (Ref) |  | 1 (Ref) |  |
| Q2 | 1.27 (1.11, 1.45) | <0.001 | 1.06 (0.90, 1.24) | 0.491 | 1.25 (0.93, 1.68) | 0.147 | 0.99 (0.71, 1.39) | 0.969 |
| Q3 | 1.32 (1.13, 1.54) | <0.001 | 1 (0.85, 1.19) | 0.955 | 1.38 (1.05, 1.82) | 0.022 | 1 (0.76, 1.33) | 0.995 |
| Q4 | 1.28 (1.08, 1.51) | 0.005 | 0.86 (0.71, 1.03) | 0.1 | 1.41 (1.03, 1.92) | 0.031 | 0.92 (0.67, 1.27) | 0.604 |
| *P* value for trend |  | 0.005 |  | 0.048 |  | 0.034 |  | 0.629 |
| zMED |  |  |  |  |  |  |  |  |
| Continuous | 0.98 (0.93, 1.04) | 0.591 | 0.93 (0.87, 0.99) | 0.027 | 1.01 (0.91, 1.13) | 0.845 | 0.95 (0.84, 1.07) | 0.402 |
| Quartile |  |  |  |  |  |  |  |  |
| Q1 | 1 (Ref) |  | 1 (Ref) |  | 1 (Ref) |  | 1 (Ref) |  |
| Q2 | 1.05 (0.86, 1.29) | 0.615 | 1 (0.83, 1.20) | 0.977 | 1.07 (0.75, 1.54) | 0.71 | 0.97 (0.68, 1.37) | 0.864 |
| Q3 | 1.07 (0.87, 1.30) | 0.535 | 0.97 (0.82, 1.14) | 0.706 | 1.08 (0.74, 1.56) | 0.695 | 0.91 (0.64, 1.29) | 0.588 |
| Q4 | 0.98 (0.81, 1.19) | 0.823 | 0.84 (0.69, 1.02) | 0.084 | 1.03 (0.73, 1.45) | 0.875 | 0.85 (0.58, 1.23) | 0.377 |
| *P* value for trend |  | 0.734 |  | 0.053 |  | 0.932 |  | 0.291 |
| zMEDI |  |  |  |  |  |  |  |  |
| Continuous | 0.97 (0.92, 1.01) | 0.128 | 0.95 (0.90, 1.00) | 0.069 | 0.96 (0.88, 1.06) | 0.431 | 0.97 (0.87, 1.09) | 0.599 |
| Quartile |  |  |  |  |  |  |  |  |
| Q1 | 1 (Ref) |  | 1 (Ref) |  | 1 (Ref) |  | 1 (Ref) |  |
| Q2 | 1.45 (1.20, 1.76) | <0.001 | 1.07 (0.88, 1.29) | 0.505 | 1.59 (1.15, 2.21) | 0.005 | 1.08 (0.77, 1.50) | 0.654 |
| Q3 | 1.46 (1.21, 1.75) | <0.001 | 1.04 (0.87, 1.23) | 0.693 | 1.48 (1.03, 2.14) | 0.035 | 1.01 (0.70, 1.46) | 0.941 |
| Q4 | 1.11 (0.96, 1.29) | 0.159 | 0.97 (0.83, 1.13) | 0.704 | 1.19 (0.87, 1.63) | 0.283 | 1.06 (0.77, 1.47) | 0.708 |
| *P* value for trend |  | 0.782 |  | 0.458 |  | 0.727 |  | 0.813 |

^[[2]](#footnote-1)^

**Table S4C.** Hazard Ratios of Mortality According to different dietary indices among hypertensive adults after excluding participants who died within 3 years of follow-up.

| Variable | All-cause mortality | | | | Cardiovascular mortality | | | |
| --- | --- | --- | --- | --- | --- | --- | --- | --- |
|  | Model 1 | | Model 2 | | Model 1 | | Model 2 | |
|  | HR (95% CI) | *P* value | HR (95% CI) | *P* value | HR (95% CI) | *P* value | HR (95% CI) | *P* value |
| zAHEI |  |  |  |  |  |  |  |  |
| Continuous | 1.03 (0.97, 1.09) | 0.388 | 0.95 (0.89, 1.02) | 0.138 | 1.01 (0.90, 1.14) | 0.801 | 0.91 (0.79, 1.05) | 0.209 |
| Quartile |  |  |  |  |  |  |  |  |
| Q1 | 1 (Ref) |  | 1 (Ref) |  | 1 (Ref) |  | 1 (Ref) |  |
| Q2 | 1.35 (1.14, 1.60) | <0.001 | 1.1 (0.91, 1.34) | 0.338 | 1.31 (0.95, 1.81) | 0.1 | 1.05 (0.77, 1.45) | 0.751 |
| Q3 | 1.19 (0.99, 1.42) | 0.058 | 0.95 (0.81, 1.12) | 0.555 | 1.08 (0.77, 1.51) | 0.656 | 0.82 (0.59, 1.15) | 0.26 |
| Q4 | 1.17 (0.97, 1.41) | 0.102 | 0.92 (0.76, 1.12) | 0.41 | 1.2 (0.82, 1.75) | 0.359 | 0.9 (0.59, 1.37) | 0.622 |
| *P* value for trend |  | 0.32 |  | 0.166 |  | 0.595 |  | 0.41 |
| zDASH |  |  |  |  |  |  |  |  |
| Continuous | 1.07 (1.01, 1.14) | 0.024 | 0.93 (0.87, 1.00) | 0.055 | 1.12 (1.01, 1.25) | 0.03 | 0.94 (0.83, 1.06) | 0.334 |
| Quartile |  |  |  |  |  |  |  |  |
| Q1 | 1 (Ref) |  | 1 (Ref) |  | 1 (Ref) |  | 1 (Ref) |  |
| Q2 | 1.51 (1.23, 1.84) | <0.001 | 1.15 (0.97, 1.36) | 0.106 | 1.66 (1.16, 2.37) | 0.006 | 1.15 (0.81, 1.64) | 0.436 |
| Q3 | 1.53 (1.22, 1.90) | <0.001 | 1.07 (0.86, 1.34) | 0.548 | 1.48 (1.06, 2.08) | 0.021 | 0.97 (0.69, 1.35) | 0.837 |
| Q4 | 1.4 (1.14, 1.73) | 0.001 | 0.91 (0.74, 1.11) | 0.347 | 1.79 (1.26, 2.55) | 0.001 | 1.03 (0.71, 1.50) | 0.867 |
| *P* value for trend |  | 0.007 |  | 0.126 |  | 0.003 |  | 0.804 |
| zDII |  |  |  |  |  |  |  |  |
| Continuous | 1.15 (1.09, 1.23) | <0.001 | 1.11 (1.03, 1.19) | 0.009 | 1.13 (1.00, 1.27) | 0.054 | 1.12 (0.96, 1.30) | 0.144 |
| Quartile |  |  |  |  |  |  |  |  |
| Q1 | 1 (Ref) |  | 1 (Ref) |  | 1 (Ref) |  | 1 (Ref) |  |
| Q2 | 1.17 (0.96, 1.43) | 0.109 | 1.16 (0.95, 1.41) | 0.157 | 1.12 (0.81, 1.54) | 0.503 | 1.11 (0.81, 1.52) | 0.509 |
| Q3 | 1.19 (0.98, 1.44) | 0.086 | 1.08 (0.87, 1.33) | 0.5 | 1.19 (0.86, 1.65) | 0.282 | 1.15 (0.78, 1.70) | 0.483 |
| Q4 | 1.49 (1.27, 1.76) | <0.001 | 1.3 (1.04, 1.62) | 0.022 | 1.41 (1.02, 1.95) | 0.039 | 1.33 (0.90, 1.97) | 0.152 |
| *P* value for trend |  | <0.001 |  | 0.048 |  | 0.035 |  | 0.176 |
| zHEI-2020 |  |  |  |  |  |  |  |  |
| Continuous | 1.09 (1.02, 1.16) | 0.007 | 0.93 (0.87, 1.00) | 0.035 | 1.14 (1.01, 1.29) | 0.037 | 0.95 (0.83, 1.09) | 0.468 |
| Quartile |  |  |  |  |  |  |  |  |
| Q1 | 1 (Ref) |  | 1 (Ref) |  | 1 (Ref) |  | 1 (Ref) |  |
| Q2 | 1.26 (1.09, 1.47) | 0.002 | 1.05 (0.88, 1.26) | 0.588 | 1.09 (0.79, 1.50) | 0.587 | 0.85 (0.59, 1.22) | 0.372 |
| Q3 | 1.35 (1.15, 1.58) | <0.001 | 1.02 (0.85, 1.22) | 0.86 | 1.3 (0.99, 1.72) | 0.061 | 0.91 (0.68, 1.23) | 0.557 |
| Q4 | 1.27 (1.06, 1.53) | 0.008 | 0.83 (0.68, 1.01) | 0.068 | 1.36 (0.98, 1.90) | 0.067 | 0.82 (0.58, 1.16) | 0.273 |
| *P* value for trend |  | 0.008 |  | 0.038 |  | 0.051 |  | 0.417 |
| zMED |  |  |  |  |  |  |  |  |
| Continuous | 1 (0.94, 1.07) | 0.907 | 0.94 (0.88, 1.01) | 0.093 | 1.02 (0.91, 1.16) | 0.707 | 0.93 (0.81, 1.06) | 0.27 |
| Quartile |  |  |  |  |  |  |  |  |
| Q1 | 1 (Ref) |  | 1 (Ref) |  | 1 (Ref) |  | 1 (Ref) |  |
| Q2 | 1.04 (0.83, 1.30) | 0.722 | 0.98 (0.79, 1.21) | 0.845 | 0.98 (0.66, 1.46) | 0.938 | 0.84 (0.58, 1.23) | 0.372 |
| Q3 | 1.1 (0.88, 1.36) | 0.406 | 0.99 (0.81, 1.20) | 0.914 | 1.06 (0.71, 1.59) | 0.777 | 0.83 (0.57, 1.23) | 0.363 |
| Q4 | 1.01 (0.81, 1.25) | 0.934 | 0.84 (0.67, 1.05) | 0.133 | 1 (0.68, 1.46) | 0.995 | 0.73 (0.49, 1.09) | 0.125 |
| *P* value for trend |  | 0.892 |  | 0.108 |  | 0.9 |  | 0.126 |
| zMEDI |  |  |  |  |  |  |  |  |
| Continuous | 0.98 (0.93, 1.03) | 0.359 | 0.95 (0.90, 1.01) | 0.106 | 0.97 (0.88, 1.08) | 0.584 | 0.96 (0.84, 1.09) | 0.507 |
| Quartile |  |  |  |  |  |  |  |  |
| Q1 | 1 (Ref) |  | 1 (Ref) |  | 1 (Ref) |  | 1 (Ref) |  |
| Q2 | 1.49 (1.21, 1.84) | <0.001 | 1.09 (0.89, 1.33) | 0.422 | 1.72 (1.22, 2.43) | 0.002 | 1.16 (0.82, 1.66) | 0.405 |
| Q3 | 1.48 (1.22, 1.80) | <0.001 | 1.04 (0.86, 1.26) | 0.685 | 1.51 (1.03, 2.23) | 0.036 | 1.02 (0.70, 1.49) | 0.911 |
| Q4 | 1.15 (0.98, 1.36) | 0.088 | 0.98 (0.83, 1.16) | 0.824 | 1.23 (0.86, 1.75) | 0.252 | 1.05 (0.73, 1.52) | 0.776 |
| *P* value for trend |  | 0.502 |  | 0.533 |  | 0.734 |  | 0.977 |

^[[3]](#footnote-2)^

1. HR= hazard ratio; CI= confidence interval. Model 1 was unadjusted; Model 2 was adjusted for sex, age, race, educational level, family poverty-income ratio, marital status, smoking status, BMI, waist circumference, GGT, AST, ALT, total energy intake, diabetes, CVD, CKD, hyperlipidemia, and cancer. [↑](#footnote-ref-0)
2. HR= hazard ratio; CI= confidence interval. Model 1 was unadjusted; Model 2 was adjusted for sex, age, race, educational level, family poverty-income ratio, marital status, smoking status, BMI, waist circumference, GGT, AST, ALT, total energy intake, diabetes, CVD, CKD, hyperlipidemia, and cancer. [↑](#footnote-ref-1)
3. HR= hazard ratio; CI= confidence interval. Model 1 was unadjusted; Model 2 was adjusted for sex, age, race, educational level, family poverty-income ratio, marital status, smoking status, BMI, waist circumference, GGT, AST, ALT, total energy intake, diabetes, CVD, CKD, hyperlipidemia, and cancer. [↑](#footnote-ref-2)
