# Supplementary material for: Associations of dietary indices with risk of all-cause and cardiovascular mortality in hypertensive adults
Source: Ann Med. 2025 Nov 15;57(1):2584427. doi: 10.1080/07853890.2025.2584427 (PMC12621336; doi:10.1080/07853890.2025.2584427)
Supplement: Supplemental Material [file IANN_A_2584427_SM3071.zip › suppl_data/Table S5.docx]

**Table S5** Hazard Ratios of Mortality According to different dietary indices among hypertensive adults after excluding non-Hispanic Black participants.

| Variable | All-cause mortality | | | | Cardiovascular mortality | | | |
| --- | --- | --- | --- | --- | --- | --- | --- | --- |
|  | Model 1 | | Model 2 | | Model 1 | | Model 2 | |
|  | HR (95% CI) | *P* value | HR (95% CI) | *P* value | HR (95% CI) | *P* value | HR (95% CI) | *P* value |
| zAHEI |  |  |  |  |  |  |  |  |
| Continuous | 0.97 (0.91, 1.03) | 0.269 | 0.9 (0.84, 0.96) | 0.002 | 0.98 (0.88, 1.09) | 0.689 | 0.92 (0.81, 1.06) | 0.248 |
| Quartile |  |  |  |  |  |  |  |  |
| Q1 | 1 (Ref) |  | 1 (Ref) |  | 1 (Ref) |  | 1 (Ref) |  |
| Q2 | 1.22 (1.03, 1.45) | 0.024 | 0.99 (0.82, 1.19) | 0.934 | 1.1 (0.80, 1.50) | 0.554 | 0.89 (0.64, 1.24) | 0.498 |
| Q3 | 1.07 (0.90, 1.28) | 0.442 | 0.86 (0.75, 1.00) | 0.051 | 0.98 (0.72, 1.33) | 0.882 | 0.79 (0.59, 1.07) | 0.127 |
| Q4 | 0.97 (0.80, 1.17) | 0.762 | 0.79 (0.66, 0.94) | 0.009 | 1.03 (0.72, 1.46) | 0.888 | 0.87 (0.60, 1.27) | 0.464 |
| *P* value for trend |  | 0.4 |  | 0.004 |  | 0.932 |  | 0.418 |
| zDASH |  |  |  |  |  |  |  |  |
| Continuous | 1.04 (0.98, 1.11) | 0.168 | 0.92 (0.86, 0.99) | 0.017 | 1.09 (0.97, 1.21) | 0.133 | 0.95 (0.84, 1.08) | 0.462 |
| Quartile |  |  |  |  |  |  |  |  |
| Q1 | 1 (Ref) |  | 1 (Ref) |  | 1 (Ref) |  | 1 (Ref) |  |
| Q2 | 1.36 (1.11, 1.67) | 0.003 | 1.01 (0.84, 1.21) | 0.897 | 1.5 (1.08, 2.09) | 0.017 | 1.06 (0.75, 1.50) | 0.749 |
| Q3 | 1.4 (1.13, 1.74) | 0.002 | 0.99 (0.80, 1.23) | 0.952 | 1.27 (0.89, 1.81) | 0.187 | 0.89 (0.62, 1.26) | 0.495 |
| Q4 | 1.29 (1.05, 1.58) | 0.016 | 0.85 (0.70, 1.04) | 0.118 | 1.55 (1.11, 2.17) | 0.01 | 0.98 (0.68, 1.42) | 0.926 |
| *P* value for trend |  | 0.049 |  | 0.068 |  | 0.051 |  | 0.735 |
| zDII |  |  |  |  |  |  |  |  |
| Continuous | 1.24 (1.16, 1.32) | <0.001 | 1.15 (1.06, 1.24) | <0.001 | 1.18 (1.05, 1.34) | 0.006 | 1.06 (0.92, 1.24) | 0.413 |
| Quartile |  |  |  |  |  |  |  |  |
| Q1 | 1 (Ref) |  | 1 (Ref) |  | 1 (Ref) |  | 1 (Ref) |  |
| Q2 | 1.2 (1.00, 1.45) | 0.054 | 1.13 (0.94, 1.36) | 0.18 | 1.21 (0.87, 1.68) | 0.254 | 1.1 (0.81, 1.49) | 0.555 |
| Q3 | 1.33 (1.11, 1.58) | 0.002 | 1.14 (0.94, 1.38) | 0.184 | 1.32 (0.96, 1.82) | 0.091 | 1.08 (0.73, 1.58) | 0.706 |
| Q4 | 1.75 (1.47, 2.08) | <0.001 | 1.4 (1.12, 1.75) | 0.003 | 1.62 (1.19, 2.21) | 0.002 | 1.21 (0.81, 1.79) | 0.352 |
| *P* value for trend |  | <0.001 |  | 0.004 |  | 0.002 |  | 0.415 |
| zHEI-2020 |  |  |  |  |  |  |  |  |
| Continuous | 1.05 (0.99, 1.12) | 0.11 | 0.91 (0.85, 0.97) | 0.006 | 1.14 (1.00, 1.29) | 0.048 | 0.98 (0.85, 1.13) | 0.804 |
| Quartile |  |  |  |  |  |  |  |  |
| Q1 | 1 (Ref) |  | 1 (Ref) |  | 1 (Ref) |  | 1 (Ref) |  |
| Q2 | 1.17 (1.01, 1.35) | 0.042 | 0.98 (0.82, 1.16) | 0.808 | 1.17 (0.85, 1.60) | 0.343 | 0.96 (0.67, 1.36) | 0.803 |
| Q3 | 1.3 (1.11, 1.53) | 0.001 | 0.98 (0.83, 1.17) | 0.857 | 1.33 (0.97, 1.83) | 0.081 | 0.97 (0.69, 1.36) | 0.877 |
| Q4 | 1.17 (0.97, 1.40) | 0.093 | 0.8 (0.66, 0.96) | 0.018 | 1.33 (0.94, 1.88) | 0.104 | 0.9 (0.63, 1.27) | 0.548 |
| *P* value for trend |  | 0.053 |  | 0.012 |  | 0.09 |  | 0.606 |
| zMED |  |  |  |  |  |  |  |  |
| Continuous | 0.95 (0.89, 1.01) | 0.087 | 0.89 (0.84, 0.95) | <0.001 | 0.99 (0.88, 1.12) | 0.876 | 0.94 (0.82, 1.07) | 0.367 |
| Quartile |  |  |  |  |  |  |  |  |
| Q1 | 1 (Ref) |  | 1 (Ref) |  | 1 (Ref) |  | 1 (Ref) |  |
| Q2 | 0.91 (0.76, 1.10) | 0.354 | 0.83 (0.70, 0.98) | 0.032 | 0.91 (0.64, 1.28) | 0.583 | 0.81 (0.58, 1.14) | 0.23 |
| Q3 | 0.92 (0.77, 1.11) | 0.393 | 0.82 (0.70, 0.95) | 0.01 | 0.93 (0.65, 1.35) | 0.718 | 0.79 (0.55, 1.12) | 0.189 |
| Q4 | 0.84 (0.69, 1.02) | 0.08 | 0.71 (0.59, 0.86) | <0.001 | 0.9 (0.62, 1.29) | 0.554 | 0.75 (0.51, 1.11) | 0.156 |
| *P* value for trend |  | 0.098 |  | 0.001 |  | 0.634 |  | 0.195 |
| zMEDI |  |  |  |  |  |  |  |  |
| Continuous | 0.94 (0.89, 0.98) | 0.01 | 0.93 (0.88, 0.99) | 0.019 | 0.92 (0.83, 1.02) | 0.122 | 0.94 (0.83, 1.06) | 0.29 |
| Quartile |  |  |  |  |  |  |  |  |
| Q1 | 1 (Ref) |  | 1 (Ref) |  | 1 (Ref) |  | 1 (Ref) |  |
| Q2 | 1.55 (1.26, 1.91) | <0.001 | 1.16 (0.96, 1.40) | 0.129 | 1.53 (1.05, 2.22) | 0.026 | 1.04 (0.73, 1.49) | 0.827 |
| Q3 | 1.43 (1.18, 1.75) | <0.001 | 1.02 (0.86, 1.22) | 0.801 | 1.33 (0.89, 2.00) | 0.17 | 0.89 (0.61, 1.31) | 0.56 |
| Q4 | 1.08 (0.91, 1.29) | 0.378 | 0.98 (0.83, 1.16) | 0.8 | 1.04 (0.72, 1.48) | 0.846 | 0.95 (0.67, 1.35) | 0.769 |
| *P* value for trend |  | 0.544 |  | 0.307 |  | 0.56 |  | 0.592 |

^[[1]](#footnote-0)^

1. HR= hazard ratio; CI= confidence interval. Model 1 was unadjusted; Model 2 was adjusted for sex, age, race, educational level, family poverty-income ratio, marital status, smoking status, BMI, waist circumference, GGT, AST, ALT, total energy intake, diabetes, CVD, CKD, hyperlipidemia, and cancer. [↑](#footnote-ref-0)
