# Supplementary material for: Associations of dietary indices with risk of all-cause and cardiovascular mortality in hypertensive adults
Source: Ann Med. 2025 Nov 15;57(1):2584427. doi: 10.1080/07853890.2025.2584427 (PMC12621336; doi:10.1080/07853890.2025.2584427)
Supplement: Supplemental Material [file IANN_A_2584427_SM3071.zip › suppl_data/Table S6.docx]

**Table S6.** Hazard Ratios of Mortality According to different dietary indices among hypertensive adults after excluding participants with implausible total energy intake.

| Variable | All-cause mortality | | | | Cardiovascular mortality | | | |
| --- | --- | --- | --- | --- | --- | --- | --- | --- |
|  | Model 1 | | Model 2 | | Model 1 | | Model 2 | |
|  | HR (95% CI) | *P* value | HR (95% CI) | *P* value | HR (95% CI) | *P* value | HR (95% CI) | *P* value |
| zAHEI |  |  |  |  |  |  |  |  |
| Continuous | 1.01 (0.95, 1.08) | 0.653 | 0.9 (0.84, 0.96) | 0.002 | 1.01 (0.90, 1.13) | 0.82 | 0.92 (0.81, 1.05) | 0.229 |
| Quartile |  |  |  |  |  |  |  |  |
| Q1 | 1 (Ref) |  | 1 (Ref) |  | 1 (Ref) |  | 1 (Ref) |  |
| Q2 | 1.38 (1.13, 1.67) | 0.001 | 1.14 (0.92, 1.40) | 0.242 | 1.41 (1.00, 1.99) | 0.05 | 1.17 (0.82, 1.66) | 0.387 |
| Q3 | 1.22 (1.00, 1.48) | 0.045 | 0.92 (0.77, 1.09) | 0.319 | 1.11 (0.77, 1.61) | 0.566 | 0.87 (0.61, 1.25) | 0.457 |
| Q4 | 1.12 (0.93, 1.35) | 0.248 | 0.81 (0.67, 0.97) | 0.019 | 1.16 (0.79, 1.68) | 0.45 | 0.9 (0.62, 1.32) | 0.593 |
| *P* value for trend |  | 0.599 |  | 0.003 |  | 0.792 |  | 0.306 |
| zDASH |  |  |  |  |  |  |  |  |
| Continuous | 1.08 (1.02, 1.14) | 0.008 | 0.91 (0.86, 0.97) | 0.002 | 1.1 (0.99, 1.23) | 0.076 | 0.95 (0.85, 1.05) | 0.31 |
| Quartile |  |  |  |  |  |  |  |  |
| Q1 | 1 (Ref) |  | 1 (Ref) |  | 1 (Ref) |  | 1 (Ref) |  |
| Q2 | 1.36 (1.09, 1.70) | 0.006 | 1.04 (0.86, 1.27) | 0.664 | 1.79 (1.15, 2.78) | 0.009 | 1.38 (0.90, 2.12) | 0.143 |
| Q3 | 1.51 (1.21, 1.89) | <0.001 | 1.05 (0.85, 1.30) | 0.673 | 1.68 (1.15, 2.45) | 0.008 | 1.21 (0.86, 1.71) | 0.279 |
| Q4 | 1.36 (1.10, 1.68) | 0.004 | 0.82 (0.68, 0.99) | 0.035 | 1.64 (1.12, 2.42) | 0.011 | 1.04 (0.73, 1.47) | 0.827 |
| *P* value for trend |  | 0.005 |  | 0.015 |  | 0.033 |  | 0.546 |
| zDII |  |  |  |  |  |  |  |  |
| Continuous | 1.14 (1.07, 1.21) | <0.001 | 1.15 (1.07, 1.24) | <0.001 | 1.11 (0.97, 1.28) | 0.129 | 1.13 (0.96, 1.32) | 0.152 |
| Quartile |  |  |  |  |  |  |  |  |
| Q1 | 1 (Ref) |  | 1 (Ref) |  | 1 (Ref) |  | 1 (Ref) |  |
| Q2 | 1.13 (0.95, 1.35) | 0.181 | 1.11 (0.92, 1.34) | 0.266 | 1.28 (0.93, 1.77) | 0.128 | 1.23 (0.90, 1.68) | 0.192 |
| Q3 | 1.21 (1.03, 1.42) | 0.023 | 1.16 (0.97, 1.39) | 0.099 | 1.32 (0.94, 1.85) | 0.104 | 1.25 (0.86, 1.81) | 0.24 |
| Q4 | 1.45 (1.21, 1.74) | <0.001 | 1.49 (1.19, 1.87) | <0.001 | 1.45 (0.99, 2.14) | 0.058 | 1.51 (0.96, 2.36) | 0.074 |
| *P* value for trend |  | <0.001 |  | <0.001 |  | 0.047 |  | 0.094 |
| zHEI-2020 |  |  |  |  |  |  |  |  |
| Continuous | 1.07 (1.00, 1.14) | 0.038 | 0.89 (0.83, 0.95) | <0.001 | 1.09 (0.95, 1.25) | 0.201 | 0.92 (0.80, 1.06) | 0.266 |
| Quartile |  |  |  |  |  |  |  |  |
| Q1 | 1 (Ref) |  | 1 (Ref) |  | 1 (Ref) |  | 1 (Ref) |  |
| Q2 | 1.11 (0.94, 1.30) | 0.212 | 0.93 (0.78, 1.11) | 0.405 | 1.05 (0.76, 1.45) | 0.78 | 0.87 (0.61, 1.24) | 0.451 |
| Q3 | 1.28 (1.07, 1.52) | 0.006 | 0.95 (0.79, 1.14) | 0.573 | 1.25 (0.88, 1.76) | 0.209 | 0.92 (0.64, 1.31) | 0.638 |
| Q4 | 1.22 (1.02, 1.46) | 0.031 | 0.75 (0.62, 0.91) | 0.003 | 1.2 (0.84, 1.73) | 0.317 | 0.78 (0.54, 1.13) | 0.187 |
| *P* value for trend |  | 0.011 |  | 0.004 |  | 0.226 |  | 0.268 |
| zMED |  |  |  |  |  |  |  |  |
| Continuous | 0.98 (0.92, 1.05) | 0.576 | 0.89 (0.84, 0.94) | <0.001 | 0.99 (0.88, 1.13) | 0.929 | 0.91 (0.80, 1.04) | 0.167 |
| Quartile |  |  |  |  |  |  |  |  |
| Q1 | 1 (Ref) |  | 1 (Ref) |  | 1 (Ref) |  | 1 (Ref) |  |
| Q2 | 0.96 (0.78, 1.19) | 0.739 | 0.9 (0.74, 1.10) | 0.32 | 1.02 (0.67, 1.57) | 0.918 | 0.95 (0.62, 1.46) | 0.832 |
| Q3 | 0.98 (0.81, 1.20) | 0.877 | 0.84 (0.71, 1.00) | 0.049 | 1.1 (0.74, 1.64) | 0.631 | 0.93 (0.62, 1.39) | 0.724 |
| Q4 | 0.94 (0.77, 1.14) | 0.506 | 0.72 (0.60, 0.87) | <0.001 | 0.9 (0.60, 1.37) | 0.629 | 0.72 (0.47, 1.10) | 0.125 |
| *P* value for trend |  | 0.559 |  | <0.001 |  | 0.612 |  | 0.064 |
| zMEDI |  |  |  |  |  |  |  |  |
| Continuous | 0.98 (0.93, 1.03) | 0.434 | 0.94 (0.89, 1.00) | 0.051 | 0.89 (0.79, 1.00) | 0.059 | 0.86 (0.76, 0.99) | 0.034 |
| Quartile |  |  |  |  |  |  |  |  |
| Q1 | 1 (Ref) |  | 1 (Ref) |  | 1 (Ref) |  | 1 (Ref) |  |
| Q2 | 1.45 (1.18, 1.77) | <0.001 | 1.1 (0.90, 1.33) | 0.349 | 1.47 (1.01, 2.12) | 0.042 | 1.05 (0.73, 1.51) | 0.8 |
| Q3 | 1.38 (1.13, 1.69) | 0.001 | 1.05 (0.87, 1.26) | 0.597 | 1.25 (0.83, 1.90) | 0.282 | 0.94 (0.62, 1.40) | 0.745 |
| Q4 | 1.12 (0.93, 1.35) | 0.213 | 0.96 (0.79, 1.16) | 0.668 | 0.88 (0.60, 1.28) | 0.497 | 0.77 (0.53, 1.12) | 0.171 |
| *P* value for trend |  | 0.632 |  | 0.437 |  | 0.19 |  | 0.097 |

^[[1]](#footnote-0)^

1. HR= hazard ratio; CI= confidence interval. Model 1 was unadjusted; Model 2 was adjusted for sex, age, race, educational level, family poverty-income ratio, marital status, smoking status, BMI, waist circumference, GGT, AST, ALT, total energy intake, diabetes, CVD, CKD, hyperlipidemia, and cancer. [↑](#footnote-ref-0)
