# Supplementary figures and images for: Associations of dietary indices with risk of all-cause and cardiovascular mortality in hypertensive adults
Source: Ann Med. 2025 Nov 15;57(1):2584427. doi: 10.1080/07853890.2025.2584427 (PMC12621336; doi:10.1080/07853890.2025.2584427)

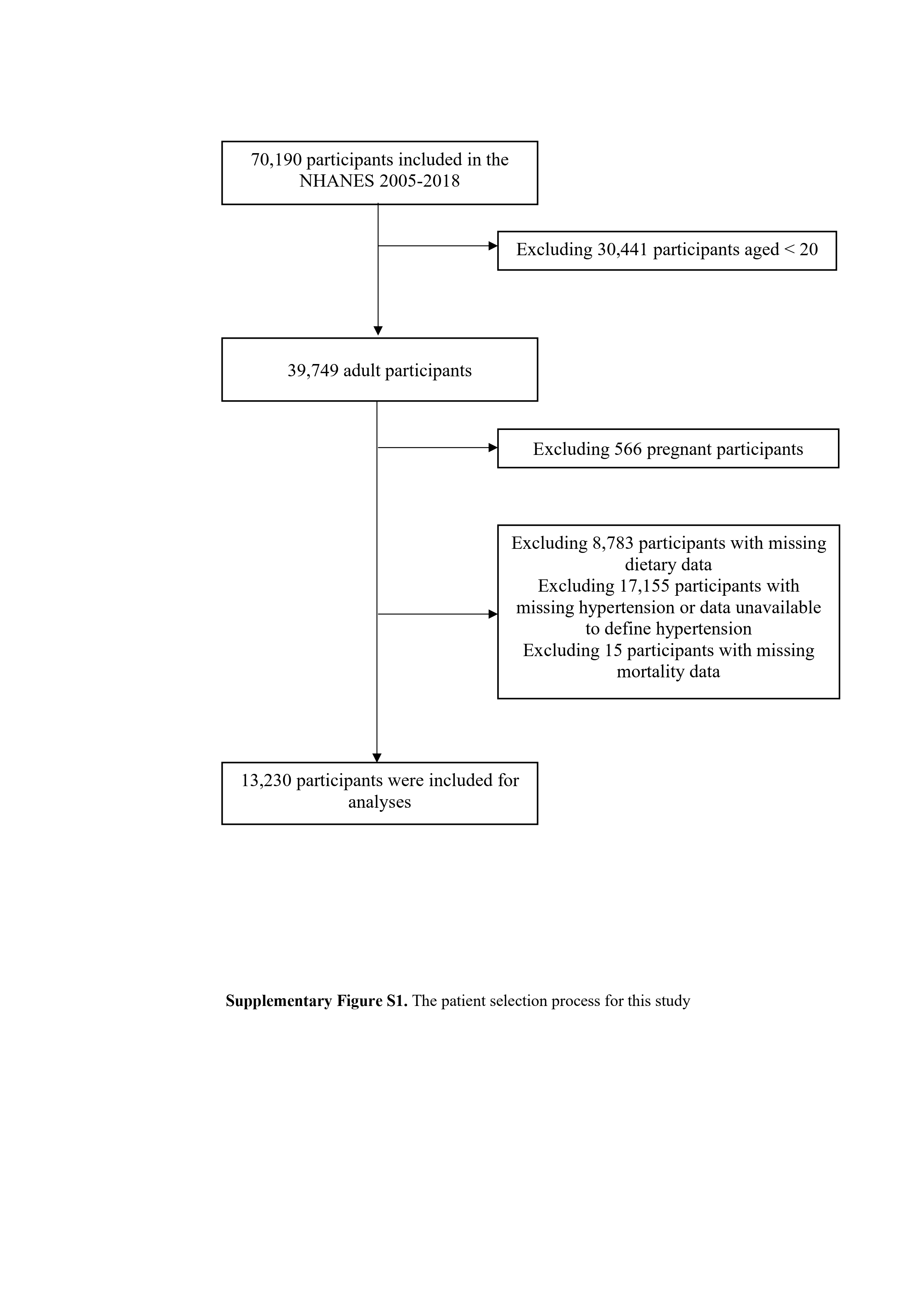

Supplement: Supplemental Material [file IANN_A_2584427_SM3071.zip › suppl_data/Figure S1.jpg]

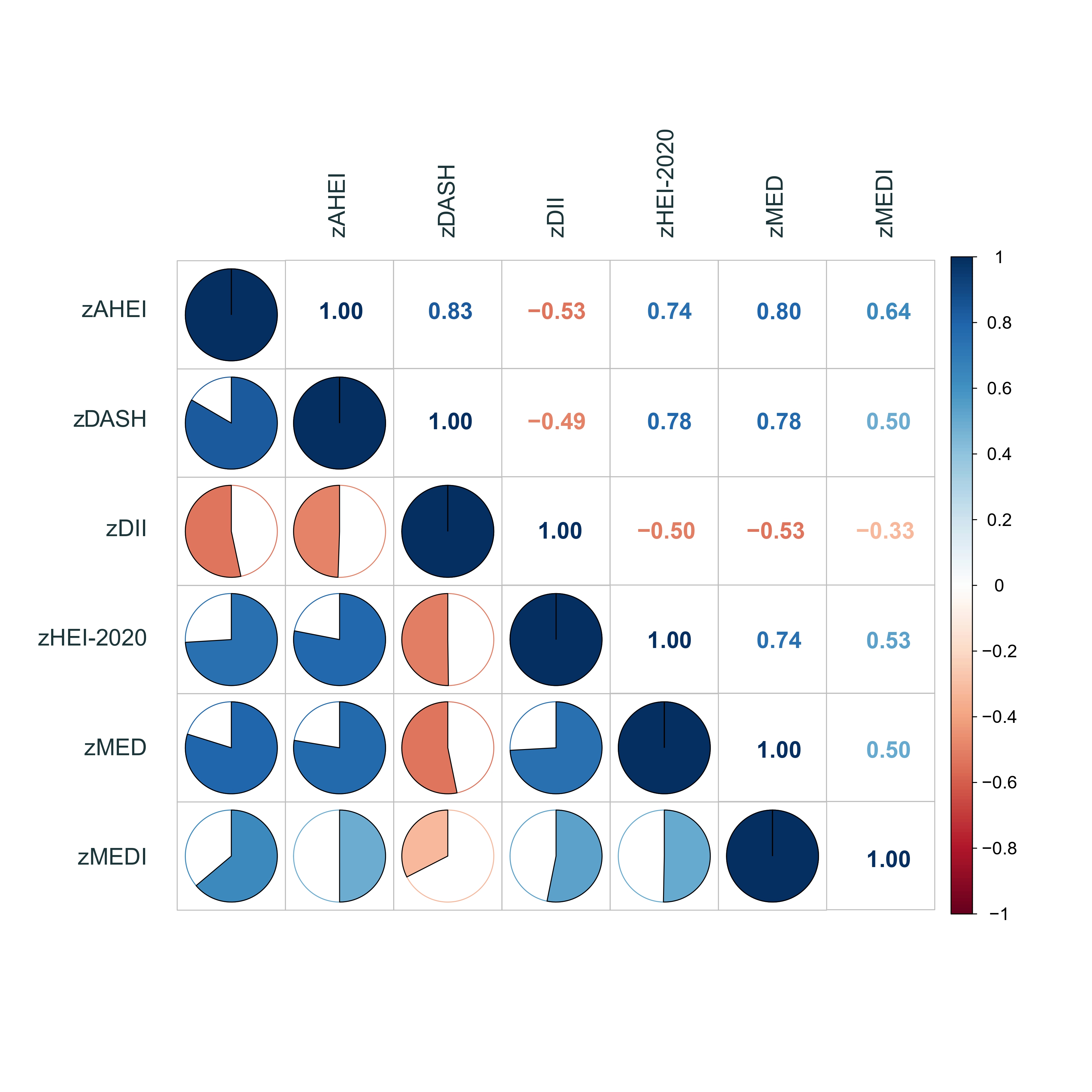

Supplement: Supplemental Material [file IANN_A_2584427_SM3071.zip › suppl_data/Figure S2.jpg]

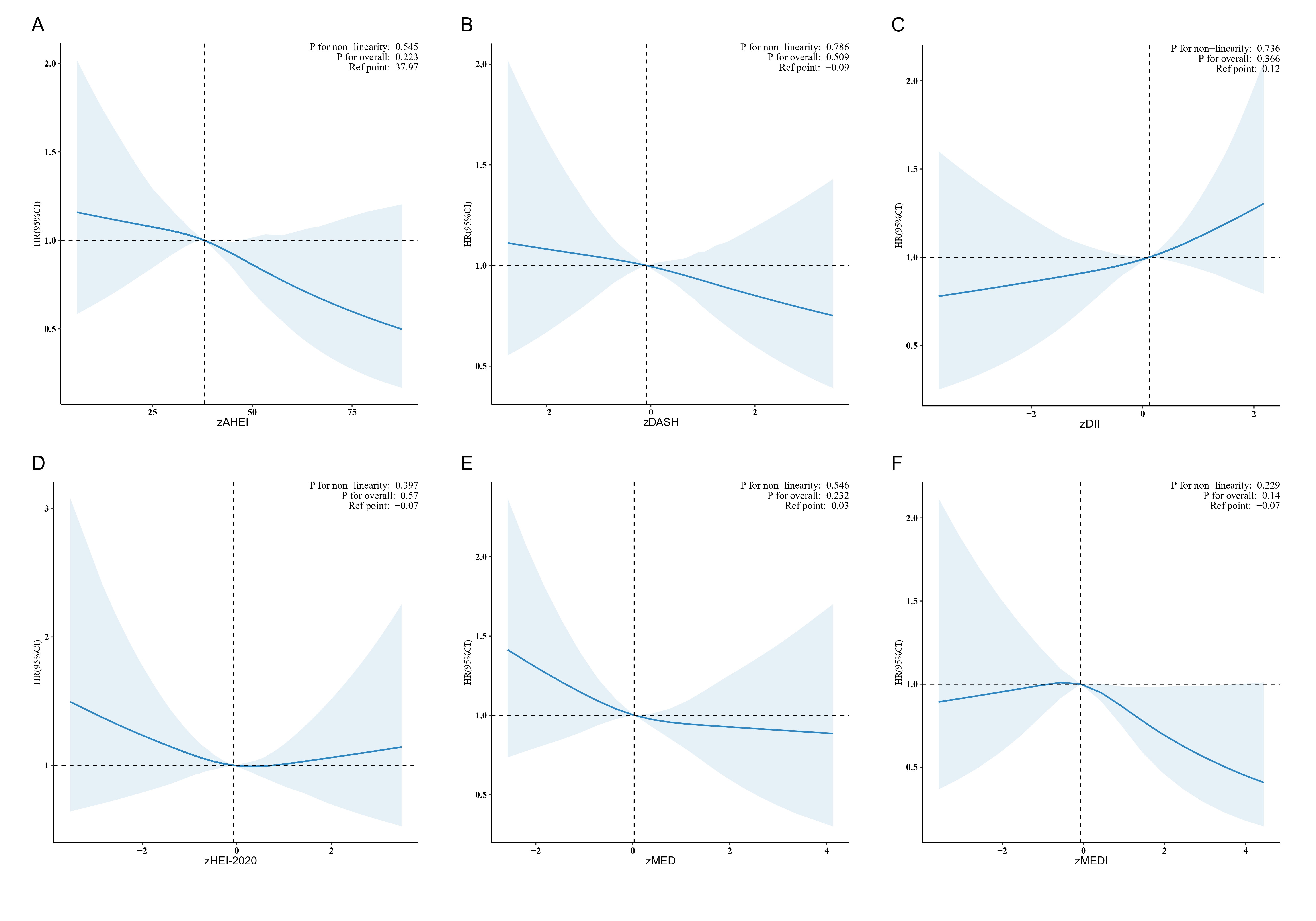

Supplement: Supplemental Material [file IANN_A_2584427_SM3071.zip › suppl_data/Figure S3.jpg]

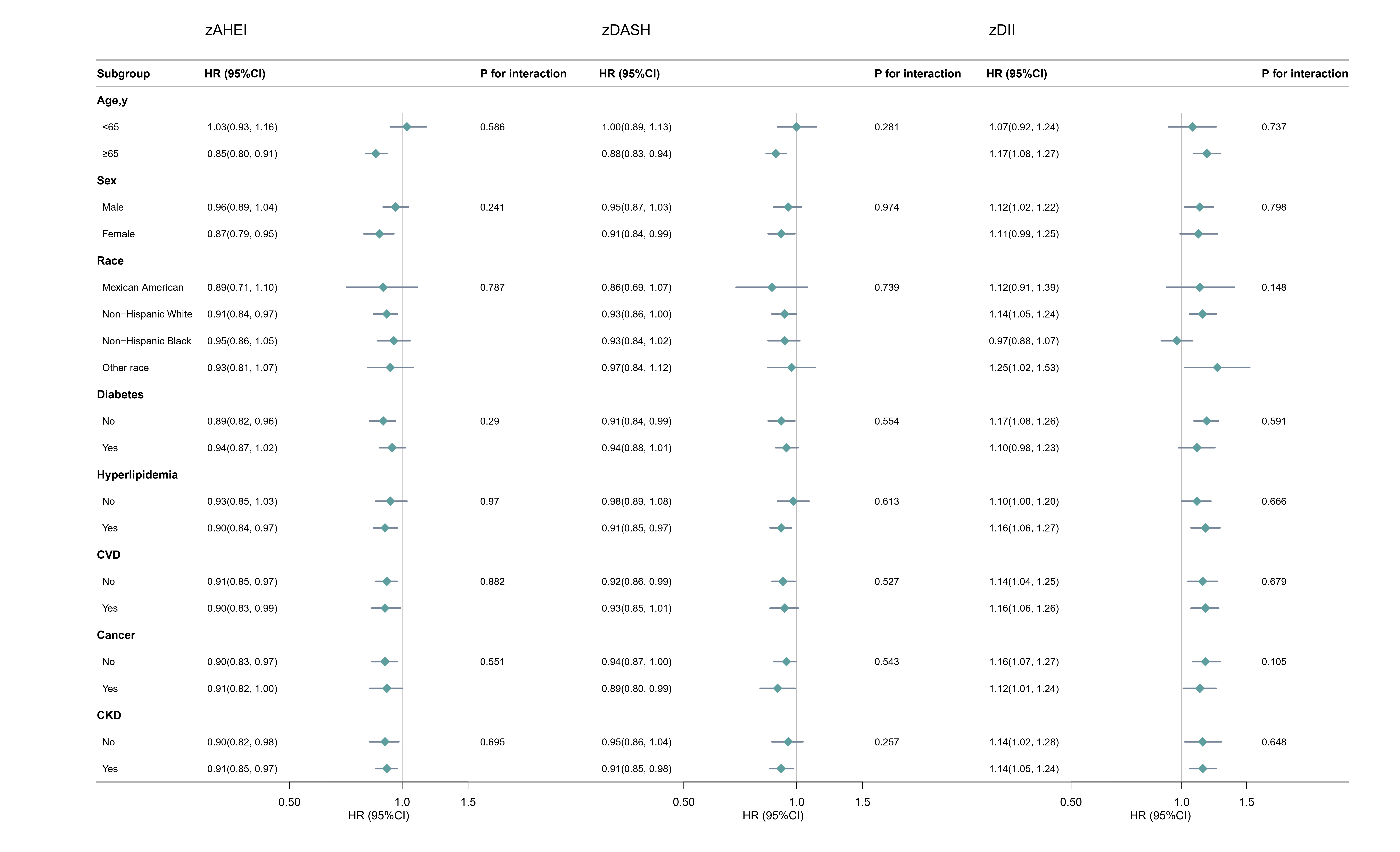

Supplement: Supplemental Material [file IANN_A_2584427_SM3071.zip › suppl_data/Figure S4.jpg]

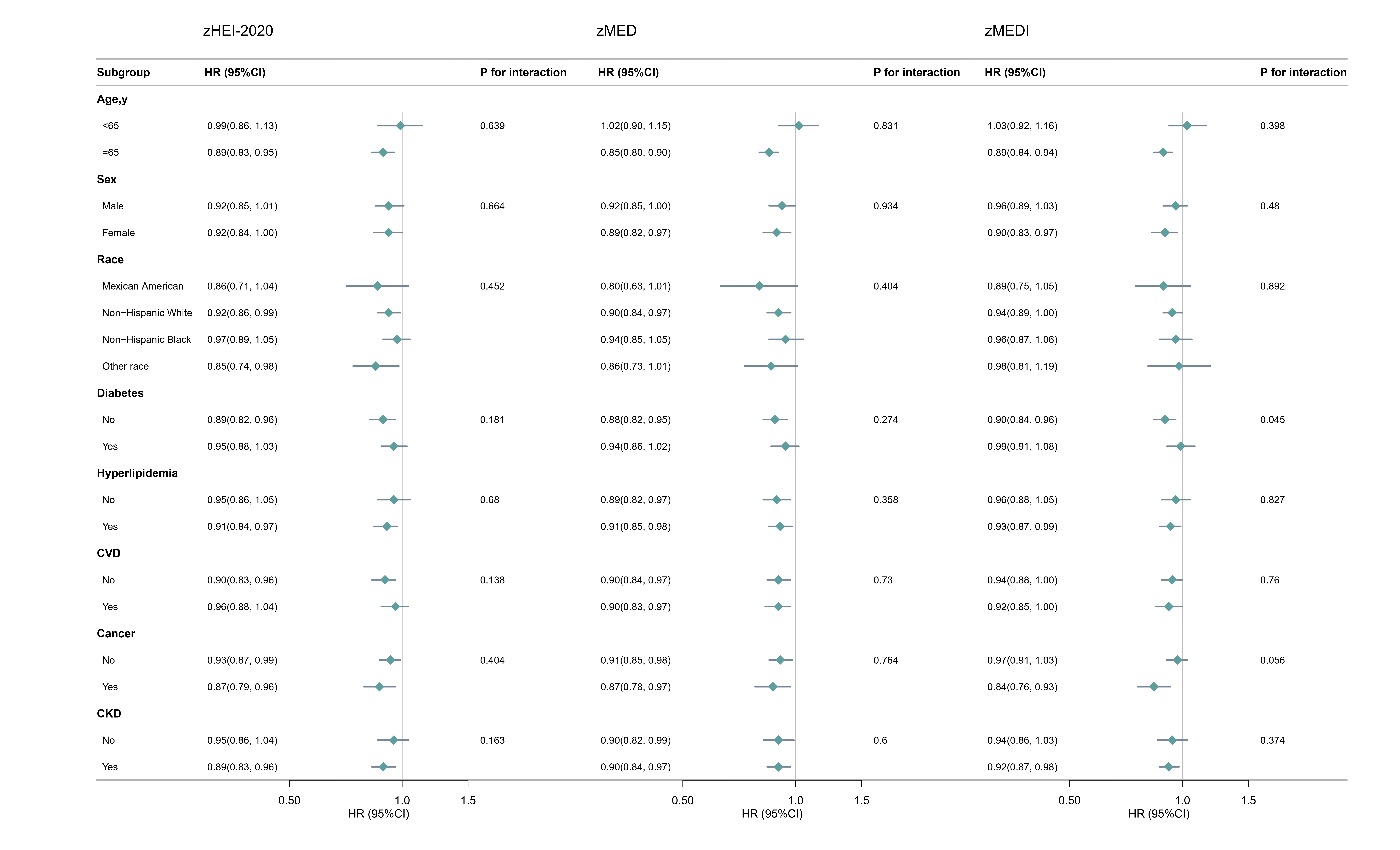

Supplement: Supplemental Material [file IANN_A_2584427_SM3071.zip › suppl_data/Figure S5.jpg]

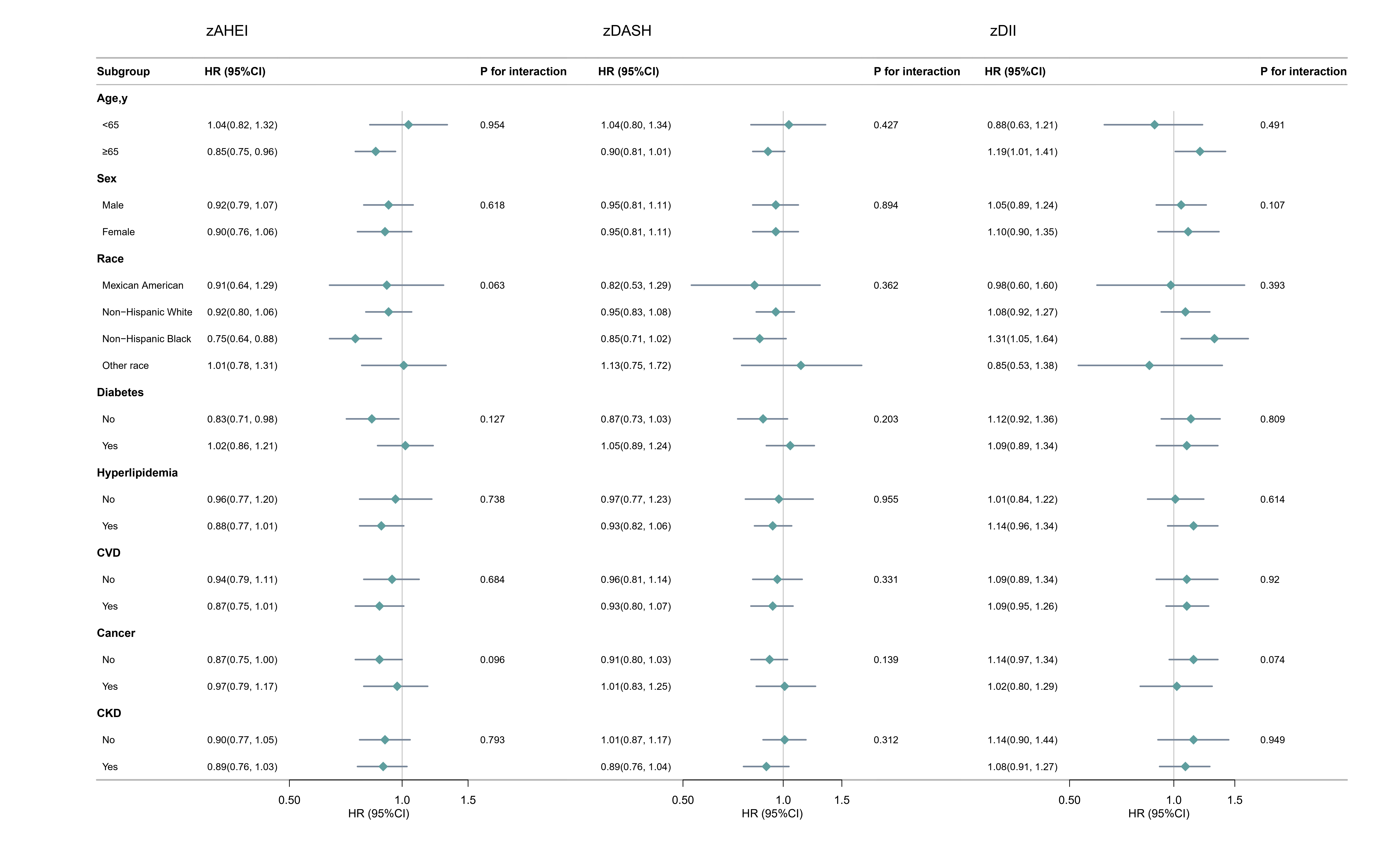

Supplement: Supplemental Material [file IANN_A_2584427_SM3071.zip › suppl_data/Figure S6.jpg]

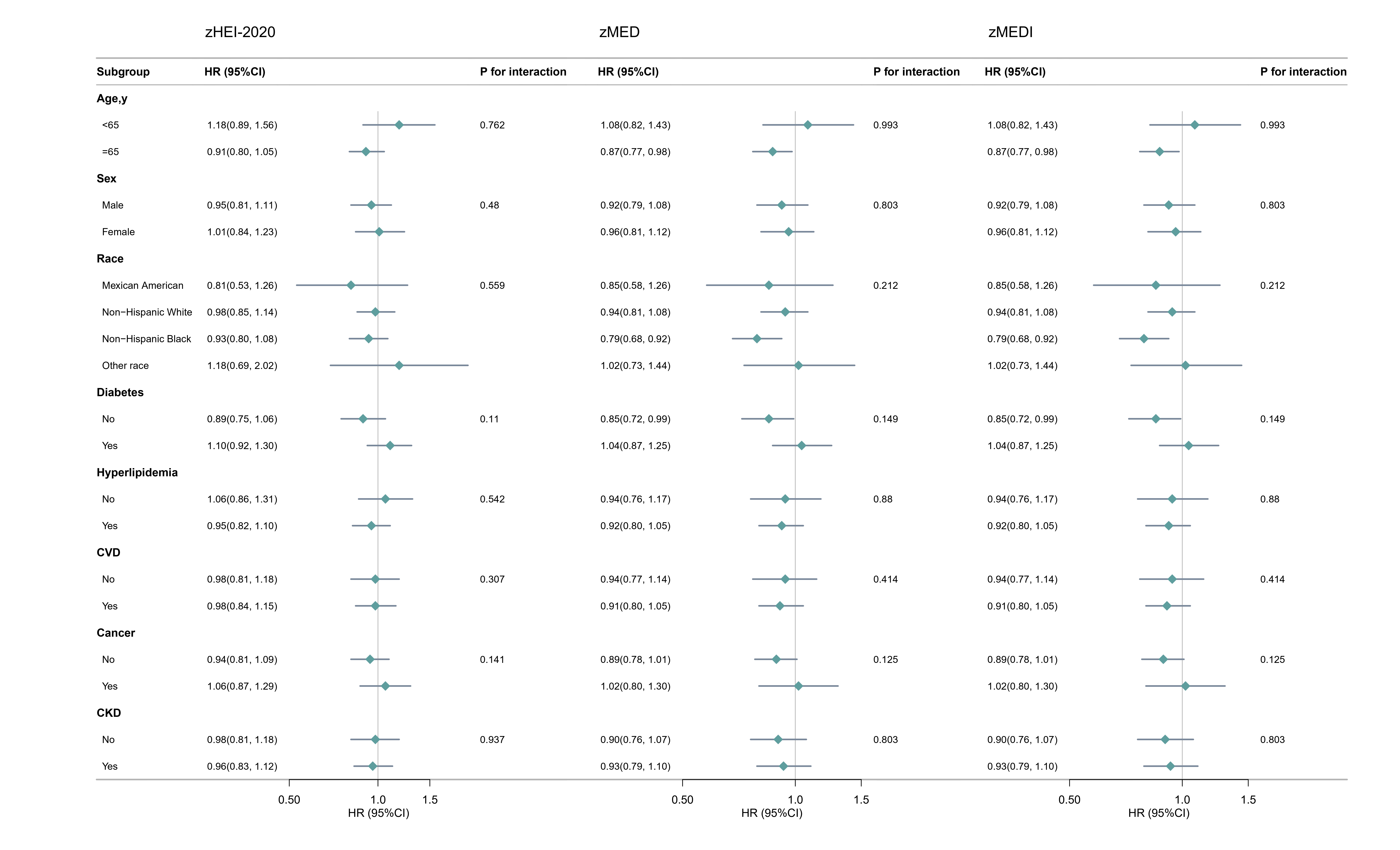

Supplement: Supplemental Material [file IANN_A_2584427_SM3071.zip › suppl_data/Figure S7.jpg]

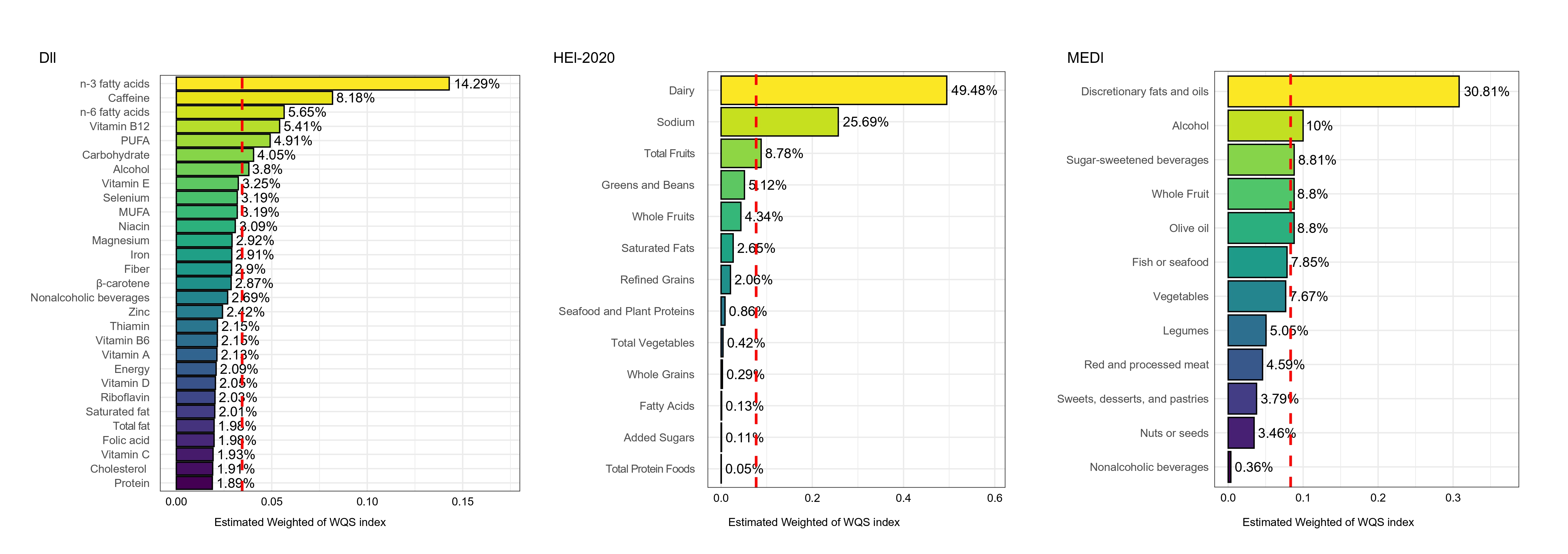

Supplement: Supplemental Material [file IANN_A_2584427_SM3071.zip › suppl_data/Figure S8.jpg]

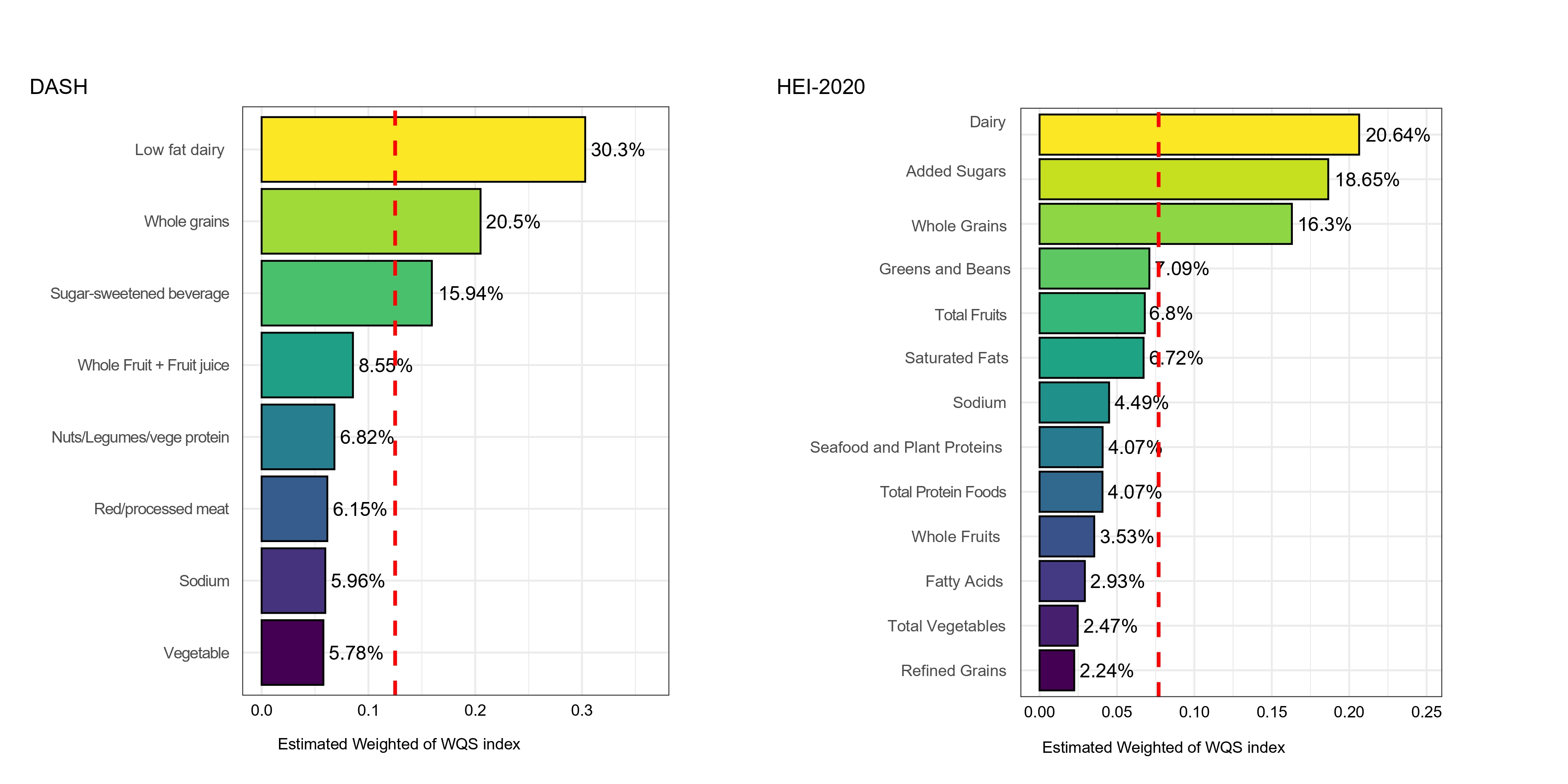

Supplement: Supplemental Material [file IANN_A_2584427_SM3071.zip › suppl_data/Figure S9.jpg]
